# Efficacy and safety of anakinra and canakinumab in PSTPIP1-associated inflammatory diseases: a comprehensive scoping review

**DOI:** 10.3389/fimmu.2023.1339337

**Published:** 2024-01-08

**Authors:** Juan Luis Sanz-Cabanillas, Francisco Gómez-García, Pedro Jesús Gómez-Arias, Ana Montilla-López, Jesús Gay-Mimbrera, Juan Ruano, Beatriz Isla-Tejera, Esmeralda Parra-Peralbo

**Affiliations:** ^1^ Inflammatory Immune-mediated Chronic Skin Diseases’ Laboratory, IMIBIC/Reina Sofia University Hospital/University of Cordoba, Córdoba, Spain; ^2^ Department of Dermatology, Reina Sofia University Hospital, Córdoba, Spain; ^3^ School of Medicine and Nursing, University of Cordoba, Córdoba, Spain; ^4^ Department of Pharmacology, Reina Sofia University Hospital, Córdoba, Spain; ^5^ Department of Pharmacy and Nutrition, Faculty of Biomedical Science and Health, Universidad Europea, Madrid, Spain

**Keywords:** autoinflammatory diseases, PSTPIP1 gene mutations, PAID, interleukin 1 pathway, IL-1 inhibitors, anakinra, canakinumab, scoping review

## Abstract

**Introduction:**

This scoping review explores the effectiveness of IL-1 pathway inhibitors in managing PSTPIP1-associated inflammatory diseases (PAID). These diseases are marked by abnormal IL-1 pathway activation due to genetic mutations.

**Methods:**

Our methodology adhered to a pre-published protocol and involved a thorough search of MEDLINE and EMBASE databases up to February 2022, following the Joanna Briggs Institute Reviewer’s Manual and the PRISMA Extension for Scoping Reviews. The review included studies reporting on IL-1 pathway inhibitor use in PAID patients.

**Results:**

From an initial pool of 5,225 articles, 36 studies involving 43 patients were selected. The studies predominantly used observational designs and exhibited diversity in patient demographics, treatment approaches, and outcomes. Anakinra and canakinumab demonstrated promise in treating sterile pyogenic arthritis, pyoderma gangrenosum, and acne (PAPA) and PSTPIP1-associated myeloid-related-proteinemia inflammatory (PAMI) syndromes, with scant data on other syndromes. Notably, there was a paucity of information on the adverse effects of these treatments, necessitating cautious interpretation of their safety profile.

**Conclusion:**

Current evidence on IL-1 pathway inhibitors for PAID is primarily from observational studies and remains limited. Rigorous research with larger patient cohorts is imperative for more definitive conclusions. Collaborative efforts among specialized research centers and international health initiatives are key to advancing this field.

## Introduction

Autoinflammatory syndromes are marked by an innate immune response dysregulation, leading to recurrent systemic inflammation episodes (1). Central to this process are mutations in the PSTPIP1 gene, which lead to the accumulation of intracellular triggers that exacerbate cellular stress and amplify the activation of inflammasome sensors (2, 3). Inflammasomes are cytosolic protein complexes that play a crucial role in producing key proinflammatory cytokines like interleukin-1β (IL-1β) and IL-18 through caspase 1 activation (4, 5).

The PSTPIP1 gene encodes the proline-serine-threonine phosphatase-interacting protein 1, predominantly expressed in hematopoietic tissues. This protein is essential in cytoskeletal organization and inflammatory responses. It consists of four distinct domains: the N-terminal FER-CIP4 homology domain, the cdc15-like segment, a PEST-rich region, and the C-terminal SH3 domain. Notably, the coiled-coil region within the cdc15-like segment and the C-terminal SH3 domain are critical for binding to pyrin, an inflammasome sensor (6, 7).

One of the earliest identified PAID was sterile pyogenic arthritis (PA), pyoderma gangrenosum (PG), and acne (PAPA) syndrome (Orphan ID: 69126), first described in 1997 and linked to chromosome 15 (5). PAPA syndrome is characterized by its namesake symptoms and shows high variability in its manifestation and genetic penetrance, often associated with various heterozygous mutations in the PSTPIP1 gene (8, 9).

In 2012, Braun-Falco et al. reported a related condition, PASH syndrome (Orphan ID: 289478), involving PG, acne, and hidradenitis suppurativa (HS) (10). This syndrome is linked to alleles with a high number of CCTG motif repeats near the PSTPIP1 promoter and other mutations in the gene (11). Moreover, variations in PSTPIP1 have been connected to other syndromes, including PAPASH syndrome (Orphan ID: 641380), PsAPASH syndrome, and PSTPIP1-associated myeloid-related-proteinemia inflammatory (PAMI) syndrome (Orphan ID: 251523) (12, 13), as outlined in **Table 1**. PG is a common clinical manifestation among all these syndromes, **Table 1**. The mutations causing PAPA, as well as in most of the other PAID syndromes, lead to a gain of function of the PSTPIP1 protein. Interestingly, there are mutations in this same gene, caused by aberrant splicing, that result in a partial, if not any, lack of function of the protein and leads to sporadic PG alone (12). However, IL-1b is upregulated in both, sporadic and syndromic PG (13).

**Table 1:** Genetics and clinical manifestations of the different PAID syndromes.

Full name	Syndrome (ORPHAN ID)	First Reported Case	Clinical Manifestations								Syndrome-Associated PSPTIP1 Mutations	Affected PSPTIP1 Protein Domain
			PA	A	PG	SH	SA	PsA	UC	Additional specific signals or symptoms		
Sterile pyogenic arthritis, pyoderma gangrenosum and acne syndrome	PAPA (69126)	1997								Arthralgia, tendinitis, periostitis with osteolytic lesions, ulcers, pustulosis, abscesses, rosacea, urticaria, edema, thrombophlebitis; anemia, elevated acute phase reactants, otitis, renal involvement, and diabetes mellitus.	p.A230T p.E250Q p.E250K p.D246N p.E256G p.D266N p.G258A c.G904A	2
PSTPIP1-associated myeloid-related-proteinemia inflammatory syndrome	PAMI (251523)	2015								**Hyperzincemia, hypercalprotectinemia**, **neutropenia,** arthralgia, morning stiffness, pustular rash, abscesses, erythema multiform; splenomegaly, anemia, thrombocytopenia, growth failure, lymphadenopathy, renal involvement, liver involvement, osteomyelitis, colitis, bleeding diathesis, muscular atrophy, granular T-lymphocyte clonal proliferation, and cerebral arterial vasculopathy/vasculitis with dissecting aneurysm.	p.E250K p.E257K	2
Sterile pyogenic arthritis, pyoderma gangrenosum, acne and hidradenitis suppurativa syndrome	PAPASH (641380)	2013								Abscesses	p.E277D	2
Pyoderma gangrenosum, acne and hidradenitis suppurativa syndrome	PASH (289478)	2012								High BMI, depression, diabetes mellitus, hypertension, hepatopathy; increased leukocytes and thrombocytes count, and CRP, GGT, and serum amyloid A levels.	p.Y345C p.A405C Increased number of CCTG microsaterllite repeats in the 5’UTR of PSPTIP1 gene	4
Pyoderma gangrenosum, acne vulgaris, hidradenitis suppurativa and ankylosing spondylitis syndrome	PASS (641385)	2012								Hepatitis B infection, α-thalassemia, elevated CRP, and intermittent fever.	–	–
Psoriatic Arthritis, pyoderma gangrenosum, acne, suppurative hidradenitis	PsAPASH (641390)	2015								Psoriasis, pseudotinea amiantacea, and purulent painful nodules.	–	–
Pyoderma gangrenosum, acne and ulcerative colitis syndrome	PAC (-)	2015								Recalcitrant pustular rash	p.G403R	4

PA, pyogenic arthritis; A, acne; PG, pyoderma gangrenosusm; SH, supurative hidradenitis; SA, ankylosing spondylitis; PsA, psoriatic arthritis; UC, ulcerative Colitis; p, protein; c, cDNA; blue light colour: symptom showed by the syndrome (blue light colour).

Treating PAID syndromes is complex and typically necessitates a multidisciplinary strategy (14). A variety of treatment options have been explored, ranging from monotherapy to combination therapies. These include isotretinoin, various anti-inflammatory agents (such as non-steroidal anti-inflammatory drugs, corticosteroids, colchicine, and thalidomide), immunosuppressive agents (like methotrexate, cyclosporine, mycophenolate mofetil, tacrolimus, azathioprine, and leflunomide), tumor necrosis factor-alpha inhibitors (infliximab, etanercept, and adalimumab), as well as plasmapheresis, intravenous immunoglobulins, and allogeneic hematopoietic stem cell transplantation (15). The efficacy of these treatments varies from patient to patient, often requiring a personalized, trial-and-error approach to identify the most effective regimen for each individual.

With advancing knowledge of the pathogenesis of autoinflammatory diseases, targeted treatments have become more prevalent. IL-1β, in particular, has garnered significant attention within the IL-1 family for its crucial role in several autoinflammatory diseases (16). In a non-sensitive genetic background, the inflammasome typically activates only in response to infections, playing essential roles in pathogen defense, damaged cell removal, and adaptive immune response stimulation (17). However, in various inflammatory disorders, infectious diseases, and cancers, this regulatory balance can be disrupted (18).

PSTPIP1 interacts with several proteins, including protein-tyrosine phosphatase (PTP-PEST), the Wiskott–Aldrich syndrome protein (WASP), the c-Abl kinase, CD2, the Fas ligand (FASL), and Pyrin/TRIM20 (19, 20). The activation of Pyrin triggers the inflammasome and consequently leads to the production of inflammatory molecules, notably IL-1β and IL-18 (21). Both IL-18 and IL-1β, members of the IL-1 family, play key roles in innate immunity and are closely linked to autoinflammatory diseases (5). Specifically, IL-1β is known as a potent endogenous pyrogen and an effective recruiter and activator of neutrophils and macrophages (20).

The development of drugs specifically targeting IL-1β offers promising treatment options for autoinflammatory diseases (22, 23). These include monoclonal antibodies (mAbs) and recombinant receptor proteins fused to human immunoglobulin G (IgG) fragments. Currently, five drugs are specifically designed to target the IL-1 pathway:

a) Anakinra, a recombinant IL-1 receptor antagonist (IL-1RA), which competes with IL-1 receptor agonists for receptor binding. b) Rilonacept, acting as a soluble decoy receptor, inhibits the activation of IL-1 receptor I. c) Canakinumab, a human monoclonal antibody, selectively targets IL-1β. d) Bermekimab (MABp1), an anti-IL-1α monoclonal antibody. e) Gevokizumab, another anti-IL1-β monoclonal antibody.

The safety and efficacy of these drugs in treating immune-mediated disorders were recently reviewed by Arnold et al. (22).

Given the rarity and recent identification of PAID syndromes, there’s a notable scarcity of evidence and secondary research, like systematic reviews, on IL-1 pathway-modulating agents for their treatment (24, 25). This gap underscores the necessity of synthesizing evidence from primary studies on the use of anti-IL-1 drugs in treating PAPA, PASH, and other PAID syndromes. Such a synthesis should include mapping published articles, examining the epidemiology and genetic characteristics, and evaluating the efficacy and safety of anti-IL-1 drugs. This analysis will be crucial in identifying knowledge gaps and formulating research questions for future systematic reviews.

We aim to present and analyze the current evidence on the use of drugs targeting the Interleukin 1 Pathway in PSTPIP1-associated inflammatory diseases, specifically focusing on PAPA, PASH, PAPASH, PASS, PAMI, PAC, and PsAPASH syndromes.

## Methods

We have pre-published a scoping review protocol to guide our study (26). The conduct and reporting of our research adhered to the methodologies outlined in the Joanna Briggs Institute Reviewer’s Manual (27) and followed the PRISMA Extension for Scoping Reviews (PRISMA-ScR) guidelines (28).

### Literature searches

Strategies for literature search and eligibility criteria are described in **Supplementary Methods (Supplementary Table S1)**.

### Eligibility criteria

For inclusion in our review, papers needed to present evidence of using drugs that target the interleukin 1 pathway in PAID. Criteria for inclusion were: studies written in English, involving human participants, and addressing the conditions specified in our research question, with no restrictions on publication date or format. Exclusion criteria encompassed studies outside our conceptual framework, such as those not focused on IL-1 pathway inhibitors in the target population, animal or *in vitro* studies, articles not in English, and non-scientific reviews.

### Data charting

A data charting form was collaboratively developed by two researchers to identify the variables for extraction. This form was initially tested on five studies, with the selected variables being recorded in a.csv file. Both reviewers independently charted data from the studies, regularly discussed their findings, and iteratively updated the form. The final report includes variables related to study design and metadata extracted from primary sources. Data were primarily gathered from clinical trial webpages, supplemented by congress abstracts and full-text articles when necessary.

### Collation, summarization and reporting of results

**Figure 1:**
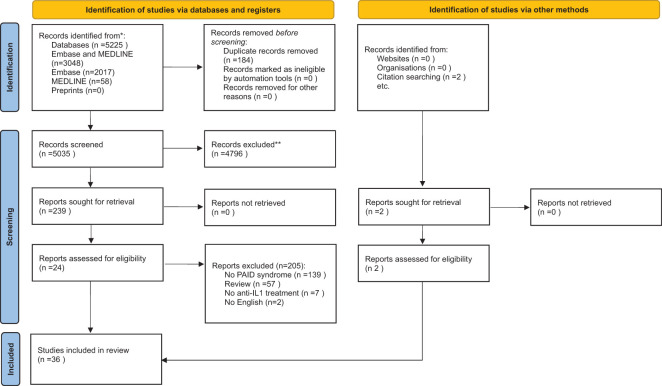
PRISMA diagram.

The results of the comprehensive research are presented using a PRISMA flow diagram (**Figure 1**). We first grouped the references and primary studies, syndrome-wise. Second, a narrative and qualitative synthesis of PAID mapping references, studies, and efficacy and safety data findings were elaborated using tables.

### Deviations from the original scoping review protocol

In conducting this scoping review, we adhered to the protocol as outlined in our published methodology in *BMJ Open* (26). No deviations from the original protocol were made during the review process. The data charting, collation, summarization, and reporting followed the predetermined methods and variables specified in the protocol.

### Compliance with ethics guidelines

This article is a review based on previously published studies and does not include any original research involving human participants or animals. Therefore, no ethical approval was required for this study.

## Results

### Searches

In February 2022, we conducted an extensive literature search on the use of anti-IL1-related drugs in autoinflammatory diseases associated with PSTPIP1 mutations, focusing on EMBASE and MEDLINE databases. This search yielded a total of 5,525 articles. After removing duplicates and conducting title, abstract, and keyword screenings, 239 studies were identified for full-text review. Ultimately, 34 articles met our inclusion criteria, and an additional two were added after reference checks of these studies (see **Figure 1** PRISMA diagram). **Supplementary Tables S2**, **S3** in the **Supplementary Material** detail the reference lists of all reviewed articles, including reasons for their inclusion or exclusion.

### Mapping studies

#### PAPA syndrome

From 2005 to 2019, 16 observational studies were published on this topic, including 11 case reports and 5 case series. These were presented as full papers (7), congress abstracts (6), or letters (3), as detailed in **Supplementary Table S4**. Notably, none of these studies followed a pre-established protocol or were registered in any public registry.

Of these studies, 11 (68.7%) were multicentric collaborations, involving an average of 7.3 authors per article (range: 3 to 14). They spanned multiple medical specialties: pediatrics and rheumatology (11 each), allergy-immunology (8), dermatology (6), internal medicine-infectious diseases (5), and others including pathology, radiology, oncology genome, laboratory medicine, and genetics (one each).

Geographically, most studies (7) originated from Italy, with others conducted in the United States, The Netherlands, Canada, Germany, Austria, and Bosnia and Herzegovina. The studies were published across various specialized journals in fields like rheumatology (11), dermatology (3), pediatrics (1), and infectious diseases (1).

Regarding funding, only two studies (12.5%) declared their sources, with one citing public funding and another disclosing both public and pharmaceutical funding. Conflicts of interest (CoIs) were declared in four studies (25%), with the most frequently cited pharmaceutical companies being Novartis, Sobi, Pfizer, Abbvie, Novimmune, Roche, and Sanofi. These disclosures are crucial for maintaining transparency about potential influences on research findings.

#### PAMI syndrome

From 2017 to 2021, we identified seven studies focusing on PAMI syndrome, all observational in nature, comprising four case reports and three case series. Notably, none adhered to a pre-established protocol or were registered in any public registry, underscoring a lack of pre-defined design and registration. Of these, five were multicenter and two unicenter, with a broad geographic distribution across Switzerland (1 study), Russia (2), Italy (3), and Brazil (1), as listed in **Supplementary Table S5**.

In terms of journal publications, the studies were diverse: one in a general medicine journal, one in allergy/immunology, two in dermatology, one in rheumatology, and two in pediatrics. The average author count was 9.14, ranging from 6 to 14. These included one full paper, one letter, three case reports, one brief report, and one abstract. The represented medical specialties were Pediatrics and Immunology (in three papers each), Dermatology, Genetics, and Rheumatology (two each), and Molecular Biology, Allergy, Nephrology, and Hematology (one each).

Funding-wise, two studies reported public funding, four declared no funding, and one did not specify. Furthermore, one study disclosed CoIs with Novartis.

#### PASH syndrome

From 2012 to 2020, seven observational case report studies on the use of anti-IL1 drugs for PASH syndrome were published, none of which followed a pre-established protocol or were registered in a public registry. Of these, five (71.4%) were unicenter studies, with two conducted in Germany and one each in Spain, France, the USA, Ireland, Australia, and Austria. All studies were published in dermatology journals, as detailed in **Supplementary Table S6**.

The publication formats varied, with three being full papers, two letters, one a case report, and one an abstract from a conference. The average number of authors was 5.29, ranging from 4 to 7. Dermatology was the most common specialty, featured in all seven papers, while rheumatology and allergy were each mentioned in one paper.

Regarding funding, most studies did not declare any, and one study provided no information on this aspect. One study disclosed a CoI with Sun Pharma, another provided no CoI information, and the remaining five declared no CoIs.

#### PASS, PAPASH and PAC syndromes

Between 2013 and 2020, we identified six studies focusing on different PAID syndromes: three investigating PASS syndrome, two examining PAPASH syndrome, and one on PAC syndrome. All studies were observational: one was a case series and the rest were case reports, as listed in **Supplementary Table S7**. Similar to previous sections, none followed a pre-established protocol or were registered in a public registry.

Geographically, two unicenter studies focused on PAC and PASS syndromes in Israel and Switzerland, respectively, while four multicenter studies on PASS and PAPASH syndromes were conducted in France and Italy. Journal-wise, five studies appeared in dermatology journals and one in a general medicine journal. The formats included three full papers (one each for PAC, PASS, and PAPASH) and three letters (one PAPASH and two PASS).

The average author count was 9, ranging from 6 to 14. Dermatology was the most common specialty, represented in all six papers, followed by Rheumatology in three, and Genetics and Pathology in two each. Other represented fields included Pediatrics, Gastroenterology, and General Internal Medicine.

Regarding funding, three studies disclosed their sources: two cited public funding, and one indicated no funding. Two studies omitted funding details. Concerning CoIs, one study disclosed multiple affiliations with pharmaceutical companies such as AbbVie, Almirall, Amgen, Boehringer-Ingelheim, Celgene, Janssen, Leo Pharma, Lilly, Mylan, Novartis, Pfizer, Sun Pharmaceuticals, Bristol-Myers Squibb, MSD, Roche-Chugai, AstraZeneca, Grunenthal, Ipsen/Menarini, Savient, Sanofi Aventis, UCB, and also reported grant support from Pfizer and L’Oreal. One paper did not provide CoI information.

### Epidemiology

#### PAPA syndrome

Data were gathered from 22 patients treated with anakinra (19 patients) or anakinra followed by canakinumab (3 patients). Aggregate data from three studies (#12, #25, and #26) on patients treated with these regimens were also included in the analysis. Out of these patients, gender information was available for 20, with 30% (six patients) being female. Geographically, the patient cohort was diverse, including seven from Italy, five from the USA, four from Canada, two each from Germany and the Netherlands, and one each from Austria and Bosnia and Herzegovina.

Arthritis emerged as the most prevalent symptom, affecting 90.1% (20 out of 22 patients). Commonly affected joints were the knees and elbows. Other symptoms observed included acne in half of the patients, with five cases being severe nodulocystic acne; PG in 45.4% (10 patients); anemia in 22.7% (5 patients); fever in 18.1% (4 patients); and leukocytosis in 13.6% (3 patients). Additional reported symptoms included lymphadenopathy, splenomegaly, thrombocytopenia, pharyngeal papillomatosis, sterile osteomyelitis, skin abscesses, recurrent otitis, pathergy, pustules, elevated CRP and ESR, abdominal pain, spontaneous abortion, oral mucosa aphthous lesions, pyogenic muscular abscess, dactylitis/tendinitis, palpebral edema, growth delay, and synovitis.

The onset age, available for 14 patients, had a median of 5 years (range: <1 to 18 years), with a median diagnosis age of 17 years (range: 2-51 years). Notably, 95.5% (21 out of 22) had received prior treatments before anti-IL1 drugs, including systemic and intralesional corticosteroids, NSAIDs, methotrexate, infliximab, tacrolimus ointment, adalimumab, cyclosporine A, isotretinoin, dapsone, antibiotics, and in some cases, gold salts, hydroxychloroquine, etanercept, colchicine, mycophenolate mofetil, plasmapheresis, and thalidomide. This diversity highlights the range of treatments attempted before IL-1 targeted therapy.

#### PAMI syndrome

Data were obtained from nine patients treated with anakinra (5 patients), anakinra followed by canakinumab (2 patients), or solely canakinumab (2 patients). Of these, three were female, five were male, and data were unavailable for one patient. Geographically, two patients each hailed from Italy and Russia, with one each from Brazil, Germany, and the United Kingdom, and data missing for one patient.

Regarding symptoms, arthritis was observed in 55.5% (5 out of 9 patients), arthralgia in 11.1% (1 patient), with data missing for two patients. Acne was present in 22.2% (2 patients), absent in 44.4% (4 patients), and data were unavailable for three patients. PG was noted in 22.2% (2 patients), absent in 55.5% (5 patients), with missing data for two. Other symptoms included elevated levels of zinc and calprotectin MRP8/14, anemia, hepatosplenomegaly, leukopenia with neutropenia, mild thrombocytopenia, osteomyelitis, trilineage dysplasia, and increased CRP and ESR.

The onset age, available for five patients, had a median of 1.2 months (range: birth to 7 years). The median diagnosis age was 4.7 years (range: 6 months to 23 years). Patients had previously received treatments including adalimumab, various antibiotics, azithromycin, ceftriaxone, colchicine, corticosteroids, cyclophosphamide, dapsone, etanercept, IVIG or HDIVIG, infliximab, intra-articular steroid injections, methylprednisolone, NSAIDs, prednisone, isotretinoin, rituximab, ruxolitinib, secukinumab, tacrolimus, tocilizumab, and topical tacrolimus.

#### PASH syndrome

Data from seven patients (three females) treated with anakinra (six patients) and canakinumab (one patient) were analyzed. These patients were from diverse locations including Russia, Spain, Turkey, Ireland, the USA, Australia, and France. All patients exhibited HS, while acne and PG were also prevalent symptoms. A high BMI was noted in 42.9% (three out of seven) of the patients. Other observed comorbidities and laboratory abnormalities included depression, diabetes mellitus, hypertension, hepatopathy, and elevated levels of leukocytes, thrombocytes, CRP, gamma-glutamyltransferase (GGT), and serum amyloid A.

The age at onset, available for five patients, had a median of 24.5 years (range: 15 to 37 years). The median age at diagnosis was 34 years (range: 22 to 59 years). Prior treatments for these patients were varied and included corticosteroids, ciclosporin, methotrexate, sulfone, antibiotics, finasteride, surgery, infliximab, adalimumab, ustekinumab, etanercept, fumaric acid, dapsone, morphine, isotretinoin, and tildrakizumab.

#### PASS, PAPASH and PAC syndromes

We collected data from five patients treated with anakinra, encompassing various syndromes. Three patients, two females from France and one male from Congo, had PASS syndrome; one female from Moldavia had PAPASH syndrome; and one male from Israel had PAC syndrome. HS was noted in all patients with PAPASH or PASS syndromes. Acne was present in four patients: one each with PAPASH and PAC, and two with PASS syndrome. All five patients exhibited PG, and all except the PAC syndrome patient had arthritis. The three PASS syndrome patients also had spondylitis (two ankylosing spondylitis, one undifferentiated spondylitis), along with other symptoms including hepatitis B infection, α-thalassemia, elevated CRP, and intermittent fever. The PAC syndrome patient showed symptoms of ulcerative colitis and a recalcitrant pustular rash.

Onset age data, available for three patients, had a median of 30 years (range: 14 to 32 years). The median age at diagnosis, obtained from five patients, was 32 years (range: 16 to 33 years). These patients had received a variety of prior treatments, including azithromycin, dapsone, etanercept, infliximab, isotretinoin, methylprednisolone, prednisolone, secukinumab, tocilizumab, topical tacrolimus, and ustekinumab.

### Genotypic variations

PAID syndromes are rare, autosomal-dominant autoinflammatory diseases characterized by incomplete penetrance and variable expression (29, 30). These genetic traits result in a spectrum of clinical manifestations linked to each mutation in the PSTPIP1 gene, complicating both the diagnosis and treatment of the disease (31).

#### PAPA syndrome

In our review, various mutations in the PSTPIP1 gene were identified among PAPA patients treated with anti-IL-1 drugs, as outlined in **Supplementary Table S8**. All cases exhibited these mutations in heterozygosity. A notable mutation was the A230T missense mutation, an alanine to threonine substitution at amino acid position 230, found in seven patients (IDs 1, 3-4, 16, 19, 29 in **Supplementary Table S2**). At amino acid position 250, two distinct substitution mutations were observed: E250Q in five patients (IDs 8, 13, 17, 19 in **Supplementary Table S2**) and E250K in two patients (IDs 17, 29 in **Supplementary Table S2**). The E256G mutation occurred in two patients (ID 17 in **Supplementary Table S2**). Additionally, a c.G904A nucleotide substitution mutation was noted in patient #8 (ID 16), while patient #10 had a wild-type allele of PSTPIP1 (ID 11 in **Supplementary Table S2**), and patient #12 had the G258A mutation (ID 6 in **Supplementary Table S2**). Unspecified mutations in PSTPIP1 were found in patients #15 (ID 2 in **Supplementary Table S2**) and patients #21 and #22 (ID 22 in **Supplementary Table S2**).

#### PAMI syndrome

For PAMI syndrome, all eight patients featured in this review shared the E250K mutation in the PSTPIP1 gene, as indicated by IDs 21 and 23 in **Supplementary Table S2**.

#### PASH syndrome

In patients with PASH syndrome, only one type of mutation in the PSTPIP1 gene was reported. This involved an increased number of CCTG microsatellite repeats in the PSTPIP1 promoter region, specifically 5 repeats on one allele and 8 on the other, indicating allelic heterogeneity (ID 5 in **Supplementary Table S2**). No additional mutations in the PSTPIP1 gene were identified for this patient.

#### PASS, PAPASH and PAC syndrome

For PASS syndrome, genetic information was unavailable for two patients (IDs 15 and 24 in **Supplementary Table S2**). The third patient (ID#4) exhibited a Q703K amino acid substitution variant in the NLRP3 gene (ID 28 in **Supplementary Table S2**). However, this NLRP3 variant is common in the general population and may also occur in asymptomatic individuals, suggesting it may be a genetic risk factor rather than the definitive cause of the syndrome. In the case of PAPASH syndrome, the single patient identified had a missense mutation in the PSTPIP1 gene, specifically E277D (ID 7 in **Supplementary Table S2**). For the patient with PAC syndrome, a G403R mutation in the PSTPIP1 gene was noted (ID 9 in **Supplementary Table S2**).

### Efficacy and Safety of IL-1 Based Agents Drugs in Treatment of PAID Syndromes

#### PAPA syndrome

We collected data for 22 patients treated with anakinra (IDs 1-4, 6, 8, 11, 13, 16-17, 19, 22, 29 in **Supplementary Table S2**) and three additional patients treated with canakinumab (IDs 6, 17, 19 in **Supplementary Table S2**), **Table 2**. Anakinra treatment duration varied from five days to 38 months, while canakinumab treatment lasted eight to nine months. The most common anakinra dosage was 100 mg/day, used in 58.3% of patients with available data (seven out of 12), with no dosage reductions required. Corticosteroids were concomitantly used in five anakinra-treated patients (IDs 2, 17, 22 in **Supplementary Table S2**), with two requiring dosage reductions.

**Table 2:** Treatment response to anti-IL1 drugs in PAPA syndrome.

ID article (IDPatient)/Cycles	DRUG/DOSE-duration	Previous treatment	Concomitant treatment	Clinical efficacy			Efficacy 6-12 weeks/ 12-24 weeks/ >24 weeks/	Adverse events/recurrence
				PA	Acne	PG		
19 (1)/1	Anakinra 100 mg/day-180 days	Corticosteroids gold salts, NSAIDs, isotretinoin	Isotretinoin	Yes (c)	No	–	NA NA NA	NA NA
19 (1)/2	Canakinumab 150mg/8weeks- NA-	Corticosteroids gold salts, NSAIDs, isotretinoin	Isotretinoin	Yes (c)	No	–	NA NA NA	NA NA
17 (2)/1	Anakinra 1,5/kg/day- 38 months	Corticosteroid metotrexate	Corticosteroid no	Yes (p)	–	–	NA NA 152 weeks	NA NA
17 (3)/1	Anakinra 2mg/kg/day 31 months	no	no	–	–	Yes (p)	NA NA 124 weeks	NA NA
17 (3)/2	Canakinumab 2-4mg/8weeks 8 months	no	no	–	–	Yes (c)	NA NA 32 weeks	NA NA
17 (4)/1	Anakinra 100mg/day 36 months	no	no	Yes (c)	Yes (c)	Yes (p)	NA NA 144 weeks	NA No
17 (5)/1	Anakinra 2mg/kg/day 21 months	Intrarticular corticosteroids	no	Yes (c)	–	–	NA NA 84 weeks	NA No
17 (6)/1	Anakinra 100mg/day 8 months	Corticosteroid metotrexate	metotrexate	Yes (c)	NA	–	NA NA 32 weeks	NA Articular flares
16 (7)/1	Anakinra 12 weeks	Corticosteroid	NA	Yes (c)	–	–	2 weeks 12 weeks NA	NA NA
16 (8)/1	Anakinra 12 weeks	Corticosteroids	NA	Yes (c)	–	–	2 weeks NA NA	NA NA
13 (9)/1	Anakinra 1 month	NSAID Corticosteroids Metotrexate Infliximab Adalimumab	NA	NA	NA	NA	NA NA NA	Hepatitis B reactivation NA
11 (10)/1	Anakinra 100mg/day 26 months	NA	NA	–	–	Yes (c)	NA NA 104 months	NA No
8 (11)/1	Anakinra 6 months	NASAID Corticosteroids	NA	Yes (c)	–	–	NA 24 weeks NA	NA NA
6 (12)/1	Anakinra 100mg/day Few days	Antibiotics Corticosteroids	NA	No	No	No	NA NA NA	Adverse reaction at injection site and sickness NA
6 (12)/2	Canakinumab 150mg/8 weeks 9 months	Antibiotics Corticosteroids	NA	Yes (c)	Yes (c)	Yes (c)	8 weeks Yes 36 weeks	NA NA
4 (13)/1	Anakinra 100mg/day 5 days	NSAID Corticosteroids	NA	Yes (c)	NA	–	5 days NA NA	Pain at injection site NA
3 (14)/1	Anakinra 100mg/day 8 months	Corticosteroids Tacrolimus ointment	NA	Yes (c)	Yes (c)	Yes (c)	5 days Yes 36 weeks	NA No
2 (15)/1	Anakinra 0,3 to 1mg/kg/day 6 months	Corticosteroids Hydroxycloroquine Methotrexate Etanercept Colchicine Tacrolimus ointment Dapsone Mycofenolate mofetil Cyclosporina A Infliximab	Corticosteroids	NA	NA	No	No No NA	Infection with MRSA No
1 (16)/1	Anakinra 1mg/kg/day 7 days	Intrarticular corticosteroids	NA	Yes (c)	NA	–	2 days NA NA	NA Yes
29 (17)/1	Anakinra	Plasmapheresis Thalidomide Dapsone Infliximab Tacrolimus	NA	No	No	No	NA NA NA	NA NA
29 (18)/1	Anakinra	Corticosteroids Etanercept Adalimumab Infliximab	NA	NA	–	NA	NA NA NA	Multiple infections No
29 (19)/1	Anakinra	Isotretinoin	NA	Yes (c)	Yes (c)	–	NA NA NA	NA NA
29 (20)/1	Anakinra	Corticosteroids Cyclosporine Tacrolimus	NA	No	–	No	NA NA NA	NA NA
22 (21)/1	Anakinra 6 weeks	NSAID Corticosteroids	Corticosteroids NSAID	Yes (c)	–	–	6 weeks NA NA	NA No
22 (22)/1	Anakinra	NSAID Corticosteroids	Corticosteroid NSAID	Yes (c)	Yes (c)	NA	NA NA NA	NA NA

PA, pyogenic arthritis; A, acne; PG, pyoderma gangrenosusm; NA, not available; -, not applicable; NSAID, Non-steroidal anti-inflammatory drugs; c, complete (dark green colour); p, partial (green light colour); No, not response (red light colour). Partial (p) and Complete (c ). Response to treatment are terms defined by the original studies.

Short-term (<12 weeks) clinical response data available for seven patients showed complete improvement in six treated with anakinra and one with canakinumab. One anakinra-treated patient did not respond well in the short term. In the medium to long term (>12 to >24 weeks), 10 patients (83.3% with available data) responded well to treatment. Regarding specific symptoms, PA improved in 82.3% (14 out of 17) of patients, acne in 62.5%, and PG in 50%, with variations in response completeness.

Among the three canakinumab-treated patients, one showed complete improvement in acne, PG, and arthritis. The second patient had a long-term response improving PG but not acne or arthritis, while the third improved in arthritis without acne development or reported PG.

For anakinra safety, reported AEs included transient injection-site reactions (2 patients), infections like hepatitis B reactivation, MRSA infection, and multiple infections, plus one sickness episode. Anakinra was discontinued in four patients due to disease flare-ups. No AEs leading to discontinuation were reported for canakinumab.

#### PAMI syndrome

Efficacy and safety data were analyzed for eight patients with PAMI syndrome, as shown in **Table 3**. Four patients received anakinra treatment (IDs 31-32, 35 in **Supplementary Table S2**), three were treated with canakinumab (IDs 21, 33-34 in **Supplementary Table S2**), and two underwent sequential treatment with anakinra followed by canakinumab (IDs 31, 34 in **Supplementary Table S2**). Data were unavailable for one patient (ID 23 in **Supplementary Table S2**).

**Table 3:** Treatment response to anti-IL1 drugs in PAMI syndrome.

ID article (IDPatient)/Cycles	Syndrome	DRUG/DOSE-duration	Previous treatment	Concomitant treatment	Clinical efficacy			Efficacy 6-12 weeks/12-24 weeks/>24 weeks/	Adverse events/Recurrence
					Acne	PG	PA		
21 (5)/1	PAMI	Canakinumab 300mg every 4 weeks	Prednisolone, topical tacrolimus, infliximab, Secukinumab, prednisone and isotretinoin	Corticosteroids, cyclosporine	NA	NA	NA	No	NA
		3 months						No	NA
								NA	
23 (6)/1	PAMI	NA	NA	NA	NA	NA	NA	NA	NA
	
31 (8)/1	PAMI	Anakinra	Infliximab, tocilizumab, HD IVIG, corticosteroids, ruxolitinib, rituximab, cyclophosphamide	NA	–	–	NA	NA	NA
31 (9)/1	PAMI	Anakinra	NA	NA	–	–	–	NA	NA
31 (9)/1		Canakinumab	NA	NA	–	–	–	NA	NA
31 (10)/1	PAMI	Anakinra	Etanercept, infliximab, corticosteroids	NA	–	–	NA	NA	NA
33 (11)/1	PAMI	Canakinumab 2mg/kg/month and 4mg/kg/month	Antibiotics, corticosteroids, IVIG, colchicine,	Cyclosporine	NA	–	Yes (c)	Yes	No
								Yes	No
								Yes (2 years)	
34 (12)/1	PAMI	Anakinra	Intra-articular steroids inyections, antibiotics, adalimumab, colchicine	NA	Yes (c)	Yes (c)	NA	Yes	NA
		2 year						NA	NA
								NA	Poor compliance
34 (12)/2		Canakinumab	Intra-articular steroids inyections, antibiotics, adalimumab, colchicine, anakinra	Corticosteroids	Yes (p)	Yes (p)	NA	Yes	Proteinuria
								Yes	Flares of acne and PG
								Yes	
34 (12)/3		Canakinumab 150mg monthly	Intra-articular steroids inyections, antibiotics, adalimumab, colchicine, anakinra, corticosteroids, tacrolimus	Tacrolimus	Yes (p)	Yes (p)	NA	Yes	NA
		1 year		Corticosteroids				Yes	NA
								Yes	
35 (13)/1	PAMI	Anakinra 3mg/kg once daily	NA	NA	NA	NA	NA	Yes	Mild transient urticarial skin reaction at the injection site
		6 months						Yes	No
								Yes (6 months)	

PA, pyogenic arthritis; A, acne; PG, pyoderma gangrenosusm; NA, not available; -, not applicable; NSAID, Non-steroidal anti-inflammatory drugs; c, complete (dark green colour); p, partial (green light colour). Partial (p) and Complete (c ). Response to treatment are terms defined by the original studies.

Anakinra treatment duration ranged from 6 months to 2 years, and canakinumab treatment lasted from 3 months to 1 year. Corticosteroids were used alongside anakinra in one patient (ID 28 in **Supplementary Table S2**) and with canakinumab in two patients (IDs 21, 34 in **Supplementary Table S2**), with one also receiving tacrolimus (ID 34 in **Supplementary Table S2**).

Two anakinra-treated patients showed good clinical responses in both short-term (<12 weeks) and long-term (>24 weeks) periods (IDs 32, 35 in **Supplementary Table S2**), with complete arthritis recovery reported for one (ID 32). Data on acne and PG responses were not available for these patients.

For canakinumab-treated patients, one exhibited a good response in both short-term and medium/long-term, with complete arthritis recovery (ID 33 in **Supplementary Table S2**), but no information on acne or PG responses was provided. Another patient showed no clinical efficacy with canakinumab alone but responded well to subsequent cyclosporine treatment, particularly in PG healing (ID 21 in **Supplementary Table S2**). AE data for canakinumab-treated patients were not fully reported.

Among patients receiving both anakinra and canakinumab, one demonstrated a good short-term response with improvements in acne and PG, but no medium/long-term response data were available (ID 34 in **Supplementary Table S2**). Information on arthritis and AEs, apart from reported proteinuria, was not provided. No efficacy or safety data were available for the other patient (ID 31 in **Supplementary Table S2**).

#### PASH syndrome

Efficacy and safety data were analyzed for seven patients with PASH syndrome, as detailed in **Table 4**. Six patients received anakinra at dosages ranging from 100 to 200 mg/day for 12 to 52 weeks, and one patient was treated with canakinumab at 150 mg/week for four weeks.

**Table 4:** Treatment response to anti-IL1 drugs in PASH syndrome.

ID article (ID Patient)/ Cycles	DRUG/ DOSIS-duration	Previous treatment	Concomitant treatment	Clinical efficacy				Efficacy 6-12 weeks/ 12-24 weeks/ >24 weeks/		Adverse events /recurrence
				SH	Acne	PG					
5 (1)/1	Anakinra 100mg/day 24 weeks	Isotretinoin 40mg	Ciclosporyne	Yes (p)	Yes (c)	Yes (c)		NA NA 24 weeks		NA NA
10 (2)/1	Anakinra 100mg/day 12 weeks	Corticosteroids, antibiotics, methotrexate, sulfone, ciclosporin, finasteride, surgery, infliximab, adalimumab, ustekinumab	NA	No	–	–		No NA NA		NA NA
14 (3)/1	Anakinra NA NA	Corticosteroids, etanercept, adalimumab, fumaric acid, cyclosporine, dapsone, antibiotics, infliximab	NA	No	No	No		NA NA NA		NA NA
18 (4)/1	Anakinra 100-200mg/day 52 weeks	Morphine, antibiotics, cyclosporine, entecavir	Prednisone, NSAID	Yes (200mg/day)	Yes (c)	Yes (c)		6 weeks NA 52 weeks		NA NA
20 (5)/1	Canakinumab 150mg weekly 4 weeks	Prednisone, adalimumab, infliximab, minocycline, dapsone, methotrexate	NA	No	No	No		No NA NA		NA NA
27 (6)/1	Anakinra NA NA	Prednisolone 15mg, methotrexate, infliximab, isotretinoin, vancomycin, clindamycin, ertapenem, tildrakizumab	NA	No	No	No		NA NA NA		NA NA
36 (7)/1	Anakinra 100mg/day 15 days	Prednisone Etanercept	NA	NA	NA	NA		NA NA NA		Sigmoid diverticulitis, exanthematous drug reaction Yes

A, acne; PG, pyoderma gangrenosusm; SH, supurative hidradenitis; NA, not available; -, not applicable; NSAID, Non-steroidal anti-inflammatory drugs; c, complete (dark green colour); p, partial (green light colour); No, not response (red light colour). Partial (p) and Complete (c ). Response to treatment are terms defined by the original studies.

Among the anakinra-treated patients, two (33.3%) showed partial improvement in PG, acne, and HS. One patient improved at week 24 (ID 5 in **Supplementary Table S2**), and the second improved at week 52 after increasing the anakinra dosage to 200 mg/day (ID 18 in **Supplementary Table S2**). Both continued anakinra treatment, with the first also receiving cyclosporine. However, three other patients on a 100 mg/day anakinra dosage did not respond and discontinued the drug (IDs 5, 10, 36 in **Supplementary Table S2**), subsequently improving with alternative treatments such as infliximab (plus dapsone and cyclosporine), corticosteroids, and tildrakizumab. One patient discontinued anakinra after 15 days due to sigmoid diverticulitis and a benign exanthematous drug reaction. No additional AEs were reported for anakinra.

The patient treated with canakinumab did not respond effectively in either short or medium to long-term analysis and later showed improvement with infliximab. No AEs were reported with canakinumab.

#### PASS, PAPASH and PAC syndromes

Efficacy and safety data were compiled for patients with PASS, PAPASH, and PAC syndromes, as outlined in **Table 5**. In PASS syndrome, three patients were treated with anakinra at 100 mg/day. Two of these patients (66.67%) responded partially or completely to the treatment (IDs 15, 28 in **Supplementary Table S2**). One patient experienced total relief from PG and partial relief from arthritis, HS, and acne but had a recurrence after stopping treatment and switched to infliximab (ID 15 in **Supplementary Table S2**). Another patient showed improvement in arthritis, PG, and acne initially but not in the long-term, also switching to infliximab (ID 28 in **Supplementary Table S2**). The third patient did not respond to anakinra (ID 24 in **Supplementary Table S2**), and no AEs were reported for these patients.

**Table 5:** Treatment response to anti-IL1 drugs in PASS, PAPASH, and PAC syndromes.

ID article (IDPatient)/ Cycles	Syndrome	DRUG/ DOSE-duration	Previous treatment	Concomitant treatment	Clinical efficacy									Efficacy 6-12 weeks/ 12-24 weeks/ >24 weeks/	Adverse events /recurrence
					SH	Acne	PG	PA	UC			AS			
15 (2)/1	PASS	Anakinra 100mg/day 4 weeks	Etanercept	NA	Yes (p)	Yes (p)	Yes	–	–			Yes (p)		Yes NA NA	NA Yes
24 (3)/1	PASS	Anakinra 100mg/day NA	Ustekinumab, tocilizumab	NA	NA	–	NA	–	–			NA		No NA NA	NA NA
28 (4)/1	PASS	Anakinra 100-200mg/day 8 months	Prednisone	Corticosteroids	No	Yes	Yes	–	–			Yes		Yes Yes No	NA NA
7 (1)/1	PAPASH	Anakinra 100mg/day NA	Azithromycin, dapsone, methylprednisolone, topical tacrolimus,	NA	Yes (p)	NA	NA	NA	–			–		Partial	Partial
9 (7)/1	PAC	Anakinra 100mg/day 24 months	Prednisolone, topical tacrolimus, infliximab, Secukinumab, prednisone and isotretinoin	Corticosteroids, Isotretinoin	–	NA	NA	–	NA			–		Partial	Total

PA, pyogenic arthritis; A, acne; PG, pyoderma gangrenosusm; SH, supurative hidradenitis; SA, ankylosing spondylitis; PsA, psoriatic arthritis; UC, ulcerative Colitis; NA, not available; -, not applicable, HDIVIG, high-dose intravenous immunoglobin; c, complete (dark green colour); p, partial (green light colour); No, not response (red light colour). Partial (p) and Complete (c ). Response to treatment are terms defined by the original studies.

For PAPASH syndrome, one patient (ID 7 in **Supplementary Table S2**) treated with 100 mg/day of anakinra showed partial improvement in HS, acne, PG, and arthritis in the short term, but medium/long-term efficacy data were not available. No AEs were reported during treatment.

In the case of PAC syndrome, one patient (ID 9 in **Supplementary Table S2**) received 100 mg/day of anakinra alongside prednisone and isotretinoin. This regimen led to total efficacy in the short and long term, fully controlling PG and improving acne, with no reported AEs.

## Discussion

### Summary of findings

To the best of our knowledge, this review represents the first extensive evaluation of the therapeutic efficacy of IL-1 pathway inhibitors, particularly anakinra and canakinumab, in treating autoinflammatory diseases linked to PSTPIP1 gene mutations. The findings suggest promising results for these agents in managing various PAID syndromes, notably in improving skin manifestations and arthritis.

Anakinra and canakinumab have been used to treat PAPA, PASH, and PAMI syndromes, while only anakinra has been utilized for PASS, PAC, and PAPASH syndromes. No evidence was found for their use in PsAPASH syndrome. PAPA syndrome, being the first reported, showed the most extensive drug use evidence. The efficacy of these treatments varies based on the specific IL-1 inhibitor, the treated syndrome, and sometimes the patient’s genetic mutation. Overall, better responses were observed in patients with PAPA and PAMI syndromes compared to those with PASH syndrome.

Anakinra’s treatment duration varied, with some patients also receiving corticosteroids. While short-term response data were inconsistent, medium to long-term results indicated good responses in 91% of patients with available data. Patients non-responsive to anakinra initially showed improvement with infliximab. Anakinra demonstrated varying degrees of clinical improvement across PASH, PAPASH, and PAC syndromes, with dosage adjustments necessary in some cases.

Canakinumab exhibited mixed results, showing good response in a patient with PAMI syndrome, but data were insufficient for other patients. Safety-wise, anakinra and canakinumab generally showed favorable profiles, with similar AEs across syndromes and rare drug withdrawals due to AEs. Anakinra’s common AE was transient injection-site reactions, while infections like hepatitis B reactivation and MRSA were also reported, highlighting the potential risk associated with immunosuppressive therapies (32). Canakinumab’s safety profile was favorable, with no discontinuation-required AEs reported. However, the limited number of patients treated with canakinumab restricts the certainty of these safety conclusions. The small sample size and retrospective nature of the analysis, along with a high degree of underreporting or absence of reporting, should be considered when interpreting these findings.

### Research gaps

#### Enhancing the evidence base and collaboration for PAID syndromes

To strengthen the treatment evidence for PAID syndromes, it is crucial to prioritize well-designed, prospective studies and adopt a coordinated, multidisciplinary approach involving specialized centers and collaborative initiatives. This approach is expected to yield improved patient care, more robust research outcomes, and better-informed treatment decisions for these rare diseases.

Currently, much of the evidence in this field comes from observational studies, case reports, and retrospective analyses, which inherently limit the study design and introduce potential biases. The lack of *a priori* experimental designs has led to lower-quality evidence, affecting the strength and reliability of the evidence base for IL-1 inhibitors in treating PAID syndromes.

Future research should focus on well-structured, prospective studies with appropriate control groups to draw more definitive conclusions about the effectiveness and safety of IL-1 inhibitors. Rigorous study designs will enhance evidence quality and increase confidence in treatment recommendations.

Enhancing drug treatment evidence for PAID syndromes requires a collaborative effort involving specialized centers for rare diseases, such as NIAMS, CNDR, CIEMA, and the National Reference Center for Autoinflammatory Diseases and Periodicity Syndromes. Platforms like the Autoinflammatory Alliance and the Eurofever registry are vital for advancing research and understanding these conditions, facilitating collaboration, data sharing, and resource pooling.

Given the high costs of treating these rare diseases, fostering public-private partnerships in research and treatment is essential. Initiatives like the International Rare Diseases Consortium (IRDiRC, www.irdirc.org) and the European Alliance for Personalized Medicine (EAPM, https://www.eapm.eu.com) highlight the benefits of collaborative efforts in managing rare diseases, providing necessary resources and support for research, treatment, and access to therapies, ultimately aiding patients with PAID syndromes.

#### Addressing disparities in healthcare for PAID syndromes in non-developed countries: challenges and opportunities

Our review reveals an underrepresentation of PAID patients from non-developed countries, highlighting healthcare disparities (33). Contributing factors include limited awareness and knowledge of PAID among healthcare professionals in these regions. Given the rarity of these diseases, clinicians may lack familiarity with their clinical presentations, diagnostic criteria, and management strategies.

A critical issue is the inadequate access to specialized care. Specialized centers and experts in PAID are predominantly located in developed countries or major urban areas, limiting access for patients in non-developed countries. This is compounded by the limited availability of specialized diagnostic tools, genetic testing, and advanced laboratory facilities.

Economic constraints further exacerbate these disparities. The affordability of diagnostic tests, medications, and long-term care poses a significant challenge, often placing these healthcare services beyond the reach of many individuals, thus impacting health outcomes.

Addressing these disparities requires further research to understand the unique challenges in diagnosing and treating rare diseases like PAID syndromes in developing countries. Efforts to improve awareness, access to diagnostic resources, and treatment availability are vital in these regions.

#### Understanding the genotype-phenotype associations in PAID syndromes: challenges and insights

In diagnosing PAID syndromes, PSTPIP1 gene sequencing has been conducted in all studied cases, though less frequently in PASH syndrome, as detailed in **Tables 1**; **Supplementary Tables S8-11**. The rarity of mutations in PASH syndrome cases might be incidental, given the small patient sample. Variants in the PSTPIP1 gene have varied interpretations in autoinflammatory diseases, from pathogenic to uncertain clinical significance, due to their incomplete penetrance and variable expression, complicating genotype-phenotype correlations.

Among the phenotypes associated with PSTPIP1 mutations, three main syndromes emerge: PAMI, PAPA, and PASH (34). In PAPA syndrome, the most commonly found variants were p.A230T and p.E250Q, previously deemed pathogenic (25). All PAMI syndrome patients reviewed exhibited the p.E250K variant, a known mutation associated with the syndrome. In the PASH case reviewed, the identified variant’s functional effect was unknown, and the patient showed a partial response to anakinra (35).

These findings underscore the complexity and variability in genotype-phenotype associations in PSTPIP1-related autoinflammatory diseases, highlighting the need for more research to clarify their implications in diagnosis and treatment. Moreover, given PSTPIP1’s potential interactions with proteins other than pyrin, alternative mechanisms beyond IL-1 signaling activation might contribute to disease pathogenesis, as suggested by Omenetti et al. (36). This is supported by Klötgen et al.’s report of canakinumab’s failure and the partial positive response to secukinumab, an IL-17A inhibitor, in a PAMI case (37).

#### Improving measurement of response in PAID syndromes

The current approach to evaluating PAID syndromes relies heavily on clinical evolution and analytical parameters, yet it lacks standardized assessment criteria or consensus protocols. This can hinder the comparability and reliability of results. Implementing tools tailored to PAID syndromes, like the Auto-Inflammatory Diseases Activity Index (AIDAI), could significantly enhance the measurement of treatment response (38). AIDAI offers a standardized framework that combines various clinical and laboratory parameters, providing a consistent and objective method to assess disease activity.

Adopting a similar approach for PAID syndromes would enable more consistent and objective measurements, improving comparisons across studies and the overall quality of evidence. Studies like Omenetti et al.’s, which utilized objective measurements to assess treatment response, underscore this point (36). Their research showed reduced IL-1β secretion by monocytes and clinical improvements following anti-IL-1 treatment, demonstrating the importance of integrating biomarkers and objective assessments in response evaluation. Long-term follow-up data also affirms the efficacy of IL-1 blockers in reducing flare frequency and normalizing acute phase reactants.

By incorporating standardized assessment tools and objective measurements, such as biomarkers, into treatment evaluations for PAID syndromes, we can improve the accuracy and reliability of outcome assessments. This advancement will not only enhance patient care but also contribute to the generation of more robust evidence regarding treatment efficacy for these conditions.

#### Evaluating the efficacy of anti-IL-1 treatment in cases with pathogenic PSTPIP1 mutations

The effectiveness of anti-IL-1 treatment in cases with pathogenic PSTPIP1 mutations requires further exploration. Notably, in PAPA and PAMI syndromes, most patients with such mutations showed either complete or partial responses to anakinra or canakinumab, particularly regarding PG, acne, and PA, as seen in long-term follow-up. However, these treatments did not resolve neutropenia in reported PAMI cases (IDs 32-34 in **Supplementary Table S2**). This finding is consistent with prior research suggesting that inhibiting the IL-1 pathway may not effectively address neutropenia in PAMI patients, though it can mitigate inflammation related to pyrin in other autoinflammatory disorders (39).

In contrast, PASH syndrome cases mostly did not respond to these treatments, with those responding showing only partial improvement at higher doses or within shorter follow-up periods. The data on PAPASH, PASS, and PAC syndromes are limited and anecdotal, with anakinra being the sole treatment used to date.

Comprehensive understanding of anti-IL-1 treatment efficacy in cases with PSTPIP1 mutations necessitates more extensive research. This will enable the collection of stronger evidence and provide insights into treatment responses across various syndromes associated with these mutations.

#### Addressing underreporting and improving safety data in drug studies

The substantial underreporting and lack of detailed safety data, including serious adverse reactions, in the analyzed studies pose significant concerns. Inadequate reporting can obscure the true safety profile of drugs, leading to their potentially inappropriate use or a failure to recognize and address critical adverse reactions (40). Addressing this challenge necessitates concerted efforts from researchers, journals, and regulatory bodies.

Implementing improved reporting practices is vital for enabling informed medical decisions about the benefits and risks of IL-1 based agents. Adhering to established case publication protocols like The CARE guidelines, which offer standardized reporting guidelines for clinical case reports, could significantly enhance the completeness and quality of safety data (41). By tackling underreporting and enhancing safety data quality, we can better understand the risks associated with IL-1 based agents and other medications, ultimately improving patient care and safety.

### Strengths and limitations

This study boasts several strengths that bolster its rigor and credibility. Key among these is the adherence to a pre-established protocol and the employment of a scoping review-specific methodology, which significantly enhance the study’s transparency and reproducibility. The research team’s prior experience in conducting similar reviews further adds to the study’s rigor, bringing valuable expertise and knowledge to the table. Notably, the independence of the working groups, indicated by the absence of external funding, implies that data collection and analysis were conducted without significant external influences, thereby enhancing the objectivity and reliability of the findings. The low incidence of reported conflicts of interest (CoIs) among the authors also strengthens the study’s credibility, suggesting minimal financial or personal biases that could influence the results’ interpretation. Collectively, these elements contribute to the robustness of the research, providing confidence in the validity of its findings and conclusions.

This study faces several notable limitations. Primarily, its reliance on observational studies, which are inherently susceptible to bias and confounding variables, makes it difficult to establish causality. Consequently, the results from these studies should be interpreted with caution. The absence of randomized controlled trials (RCTs) further limits the ability to conclusively determine the drugs’ effectiveness and safety. Another significant drawback is the inclusion of a considerable number of abstracts and letters, rather than full-length articles, which often lack detailed methodology and comprehensive results. This can hinder a thorough analysis and understanding of the data.

A critical concern is the underreporting or absence of reporting on AEs. Since AEs are essential in assessing a drug’s safety profile, their incomplete documentation can obscure a complete understanding of the potential risks. While efforts were made to obtain additional information from researchers, the study still faces the challenge of a lack of comprehensive adverse event data.

Additionally, the time constraints on the literature review process may have led to the exclusion of more recent studies, potentially omitting relevant and up-to-date research. This could affect the overall comprehensiveness and relevance of the findings. Given these limitations, it’s crucial to approach the study’s safety findings with caution and acknowledge the need for further, more robust research to provide clearer evidence regarding the safety of IL-1 based agents in treating the studied syndromes.

Considering these limitations, it is important to approach the safety findings with caution and recognize the need for further research, including well-designed RCTs and studies with robust methodologies, to provide more definitive and reliable evidence regarding the safety of IL-1 based agents in the treatment of the studied syndromes.

## Conclusions

In conclusion, while IL-1 based blockers like anakinra and canakinumab demonstrate potential in treating PAID syndromes, the current evidence has notable limitations. The predominance of observational studies in this research limits the ability to establish causality and draw firm conclusions. Additionally, the study underscores issues such as potential biases, limited data availability, and the underreporting of AEs. To overcome these challenges and provide more conclusive evidence, there is a pressing need for well-designed studies, particularly RCTs. Future research should aim to ascertain the efficacy, determine optimal dosages and treatment durations, and evaluate the long-term safety profiles of IL-1 based agents in managing PAID syndromes.

## Data availability statement

The original contributions presented in the study are included in the article/**Supplementary Material**. Further inquiries can be directed to the corresponding authors.

## Author contributions

JS: Data curation, Formal analysis, Writing – original draft. FG: Data curation, Investigation, Supervision, Writing – original draft, Methodology. PA: Writing – review & editing. AM: Writing – review & editing. JG: Writing – review & editing. JR: Investigation, Supervision, Writing – original draft, Writing – review & editing, Methodology, Resources. BI: Writing – original draft, Writing – review & editing. EP: Conceptualization, Data curation, Formal analysis, Investigation, Supervision, Writing – original draft, Writing – review & editing.
