# Supplementary material for: Efficacy and safety of anakinra and canakinumab in PSTPIP1-associated inflammatory diseases: a comprehensive scoping review
Source: Front Immunol. 2024 Jan 8;14:1339337. doi: 10.3389/fimmu.2023.1339337 (PMC10801072; doi:10.3389/fimmu.2023.1339337)
Supplement: Supplementary file 1 [file DataSheet_1.pdf]

Supplementary material

Efficacy and Safety of Anakinra and Canakinumab in PSTPIP1-associated inflammatory diseases: A Comprehensive Scoping Review

Sanz-Cabanillas JL, M.D.1,2,#, Gómez-García F, M.D., Ph.D.1,2,#; Gómez Arias PJ, M.D.1,2; Montilla-López A, M.D.1; Gay-Mimbrera J, B. Sc.1; Ruano J, M.D., M.Sc., Ph.D.1,2,; Isla-Tejera B, Pharm.D., Ph.D.3,*; Parra-Peralbo E, M.Sc., PhD 4

[1]Inflammatory Immune-mediated Chronic Skin Diseases' Laboratory, IMIBIC/Reina Sofia University Hospital/University of Cordoba, Menendez Pidal Ave, 14004, Córdoba, Spain.

[2]Department of Dermatology, Reina Sofia University Hospital, Menendez Pidal Ave, 14004, Córdoba, Spain.

[3]Department of Pharmacology, Reina Sofia University Hospital, Menendez Pidal Ave, 14004, Córdoba, Spain.

[4] Department of Pharmacy and Nutrition, Faculty of Biomedical Science and Health, Universidad Europea, Calle Tajo, s/n, 28670, Villaviciosa de Odón, Madrid

[#]Equal contributors.

SUPPLEMENTARY TABLES

Table S1. Search strategies in literature databases.

Table S2. List of included studies.

Table S3. List of excluded studies with reasons for exclusion.

Table S4. Mapping studies on PAPA.

Table S5. Mapping studies on PAMI.

Table S6. Mapping studies on PASH.

Table S7. Mapping studies on PASS, PAPASH, and PAC.

Table S8. Genetic and clinical characteristics of patients with PAPA included in the review.

Table S9. Genetic and clinical characteristics of patients with PAMI included in the review.

Table S10. Genetic and clinical characteristics of patients with PASH included in the review.

Table S11. Genetic and clinical characteristics of patients with PASS, PAPASH, and PAC included in the review.

Table S1. Search strategies in literature databases.

Databases: MEDLINE and EMBASE

Search strategy:

"Treatment AND ('interleukin 1beta' OR 'interleukin 1') AND 'skin disease' OR (('canakinumab' OR 'anakinra' OR 'rilonacept' OR mabp1) AND ('candle syndrome'/exp OR 'candle syndrome' OR (neutrophilic AND urticarial AND dermatosis) OR (undifferentiated AND autoinflammatory AND syndromes) OR (autoinflammatory AND skin AND bone AND disease) OR ('cryopyrin associated' AND periodic AND syndromes) OR (aseptic AND abscess AND syndrome) OR (acute AND febrile AND neutrophilic AND dermatosis) OR ('pyrin associated' AND autoinflammation AND with AND neutrophillic AND dermatosis) OR (deficiency AND of AND 'interleukin 1' AND receptor AND antagonist) OR (nonbacterial AND osteomyelitis AND associated AND diseases) OR (papa AND syndrome) OR (neutrophilic AND dermatoses AND in AND 'adult onset' AND autoinflammatory AND syndromes) OR (majeed AND syndrome) OR (sapho AND syndrome) OR (chronic AND recurrent AND multifocal AND osteomyelitis) OR (amicrobial AND pustulosis AND of AND the AND skin AND fold) OR (refractory AND aseptic AND abscesses AND syndrome) OR 'blau syndrome' OR (chronic AND atypical AND neutrophilic AND dermatitis AND with AND lipodystrophy AND elevated AND temperature AND syndrome) OR ('neonatal onset' AND multisystem AND inflammatory AND disease) OR (chronic AND infantile AND neurologic, AND cutaneous, AND arthritis) OR 'pash syndrome' OR 'macrophage activation syndrome' OR (neutrophilic AND panniculitis) OR 'pfapa syndrome' OR (autoinflammatory AND disease AND associated AND with AND mutations) OR (hidradenitis AND supuratica) OR 'suppurative hidradenitis' OR 'psoriasis' OR 'atopic dermatitis' OR (pyoderma AND gangrenous) OR 'pyoderma gangrenosum' OR 'skin allergy'" OR 'acne' OR (pustular AND psoriasis) OR (dissecting AND cellulitis AND of AND the AND scalp) OR (childhood AND

pustular AND dermatosis) OR (refractory AND 'anti mda5' AND clinically AND amyopathic AND dermatomyopathy) OR (autoimmune AND bullous AND dermatoses) OR (acrodermatitis AND continua AND of AND hallopeau) OR 'melanoma' OR 'schnitzler syndrome' OR ('erdheim chester' AND disease) OR (neck AND squamous AND cell AND carcinoma) OR (squamous AND cell AND carcinoma) OR 'langerhans cell histiocytosis' OR 'skin cancer' OR 'multicentric reticulohistiocytosis' OR 'poems syndrome' OR 'erythema nodosum' OR prurito OR (pigmentary AND hypertrichosis AND nonautoimmune AND 'insulin dependent' AND diabetes AND mellitus AND phid AND syndrome) OR (systemic AND sclerosis AND morphoea) OR (autosomal AND recessive AND congenital AND ichthyoses) OR 'recessive dystrophic epidermolysis bullosa' OR (chronic AND ulcers) OR 'pemphigus vulgaris' OR 'erythema elevatum diutinum' OR 'xanthelasma palpebrarum' OR 'netherton disease' OR (acute AND radiation AND dermatitis) OR (aicardi AND goutieres AND syndrome) OR 'granulomatous skin disease' OR 'scleroderma' OR 'lichen sclerosus et atrophicus' OR 'pemphigus foliaceus' OR 'ocular rosacea' OR 'amyloidosis' OR 'lupus erythematosus' OR 'sjoegren syndrome' OR (still AND disease) OR 'familial mediterranean fever' OR (cryopirin AND associated AND inflammatory AND syndrome) OR 'muckle wells' OR 'tumor necrosis factor receptor associated periodic syndrome'))) AND ('case report'/de OR 'clinical article'/de OR 'clinical trial'/de OR 'clinical trial (topic)'/de OR 'cohort analysis'/de OR 'controlled clinical trial'/de OR 'controlled study'/de OR 'drug dose comparison'/de OR 'human'/de OR 'human cell'/de OR 'human tissue'/de OR 'major clinical study'/de OR 'multicenter study'/de OR 'observational study'/de OR 'phase 2 clinical trial (topic)'/de OR 'phase 3 clinical trial (topic)'/de OR 'practice guideline'/de OR 'randomized controlled trial'/de OR 'randomized controlled trial (topic)'/de OR 'retrospective study'/de OR 'systematic review'/de)'papash syndrome' AND [01-01-1989]/sd NOT [03-02-2022]/sd) OR (('papash syndrome' AND [01-01-1989]/sd NOT [03-02-2022]/sd) OR (('papash syndrome' AND [01-01-1989]/sd NOT [03-02-2022]/sd)

OR (('pash syndrome' AND [01-01-1989]/sd NOT [03-02-2022]/sd) OR (('pass syndrome' AND [01-01-1989]/sd NOT [03-02-2022]/sd) OR (('pac syndrome' AND [01-01-1989]/sd NOT [03-02-2022]/sd)

Table S2. List of included studies.

Article ID	Article
1	Anakinra for flares of pyogenic arthritis in PAPA syndrome. Dierselhuis MP, Frenkel J, Wulffraat NM, Boelens JJ. Rheumatology (Oxford). 2005 Mar;44(3):406-8.
2	Dramatic improvement of pyoderma gangrenosum with infliximab in a patient with PAPA syndrome. Stichweh DS, Punaro M, Pascual V. Pediatr Dermatol. 2005 May-Jun;22(3):262-5.
3	Targeted treatment of pyoderma gangrenonosum in PAPA (pyogenic arthritis, pyoderma gangrenonosum and acne) syndrome with the recombinant human interleukin-1 receptor antagonist anakinra. Brenner M, Ruzicka T, Plewig G, Thomas P, Herzer P. Br J Dermatol. 2009 Nov;161(5):1199-201.
4	Variable expression and treatment of PAPA syndrome. Schellevis MA, Stoffels M, Hoppenreijs EP, Bodar E, Simon A, van der Mee[illegible] JW. Ann Rheum Dis. 2011 Jun;70(6):1168-70.
5	Pyoderma gangrenosum, acne, and suppurative hidradenitis (PASH)--a new autoinflammatory syndrome distinct from PAPA syndrome. Braun-Falco M, Kovnerystyy O, Lohse P, Ruzicka T. J Am Acad Dermatol. 2012 Mar;66(3):409-15.
6	Identification of a homozygous PSTPIP1 mutation in a patient with a PAPA-like syndrome responding to canakinumab treatment. Geusau A, Mothes-Luksch N, Nahavandi H, Pickl WF, Wise CA, Pourpak Z, Ponweiser E, Eckhart L, Sunder-Plassmann R. JAMA Dermatol. 2013 Feb;149(2):209-15.
7	Pyogenic arthritis, pyoderma gangrenosum, acne, and hidradenitis suppurativa (PAPASH): a new autoinflammatory syndrome associated with a novel mutation of the PSTPIP1 gene. Marzano AV, Trevisan V, Gattorno M, Ceccherini I, De Simone C, Crosti C. JAMA Dermatol. 2013 Jun;149(6):762-4.
8	Osteolytic lesion in PAPA syndrome responding to anti-interleukin 1 treatment. Caorsi R, Picco P, Buoncompagni A, Martini A, GattorNo M. J Rheumatol. 2014 Nov;41(11):2333-4.
9	Pyoderma gangrenosum, acne and ulcerative colitis in a patient with a novel mutation in the PSTPIP1 gene. Zeeli T, Padalon-Brauc[illegible] G, Ellenbogen E, Gat A, Sarig O, Sprecher E. Clin Exp Dermatol. 2015 Jun;40(4):367-72.
10	Two cases of severe hidradenitis suppurativa with failure of anakinra therapy. Menis D, Maroñas-Jiménez L, Delgado-Marquez AM[illegible] Postigo-Llorente C, Vanaclocha-Sebastián F. Br J Dermatol. 2015 Mar;172(3):810-1.

Article ID	Article
11	Long-term efficacy of IL-1 blockers in PAPA patients. Finetti, M., Caorsi, R., Marotto, D. et al. Pediatr Rheumatol 13 (Suppl 1), P207 (2015).
12	The phenotypic variability of PAPA syndrome: evidence from the Eurofever Registry. Caorsi, R., Marotto, D., Insalaco, A. et al. Pediatr Rheumatol 13 (Suppl 1), O8 (2015).
13	Interleukin-1 receptor antagonist treatment revealed active hepatitis B infection in a boy with PAPA syndrome. Selmanovic, V., DeBenedeti, F., Omercahić-Dizdarevic, A. et al.. Pediatr Rheumatol 13 (Suppl 1), P11 (2015).
14	Successful treatment of PASH syndrome with infliximab, cyclosporine and dapsone. Staub J, Pfannschmidt N, Strohal R, Braun-Falco M, Lohse P, Goerdt S, Leverkus M. J Eur Acad Dermatol Venereol. 2015 Nov;29(11):2243-7.
15	PASS Syndrome: An IL-1-Driven Autoinflammatory Disease. Leuenberger M, Berner J, Di Lucca J, Fischer L, Kaparos N, Conrad C, Hohl D, So A, Gilliet M. Dermatology. 2016;232(2):254-8.
16	IL-1 Blocker Therapy for Articular Flares in PAPA Syndrome. Mehul Jariwala; Jennifer Stimec; Karen Watanabe Duffy; Claudia Martinez-Rios; Lynn Spiegel; Ronald Laxer. The Journal of Rheumatology 2016; 43:6
17	Disease activity accounts for long-term efficacy of IL-1 blockers in pyogenic sterile arthritis pyoderma gangrenosum and severe acne syndrome. Omenetti A, Carta S, Caorsi R, Finetti M, Marotto D, Lattanzi B, Jorini M, DelfiNo L, Penco F, Picco P, Buoncompagni A, Martini A, Rubartelli A, Gattorno M. Rheumatology (Oxford). 2016 Jul;55(7):1325-35.
18	Treatment of pyoderma gangrenosum, acne, suppurative hidradenitis (PASH) with weight-based anakinra dosing in a Hepatitis B carrier. Jennings L, Molloy O, Quinlan C, Kelly G, O'Kane M. Int J Dermatol. 2017 Jun;56(6):e128-e129.
19	Pyogenic arthritis, pyoderma gangrenosum, and acne (PAPA) syndrome: differential diagNosis of septic arthritis by regular detection of exceedingly high synovial cell counts. Löffler W, Lohse P, Weihmayr T, Widenmayer W. Infection. 2017 Aug;45(4):395-402.
20	Non-response to Interleukin-1 Antagonist Canakinumab in Two Patients with Refractory Pyoderma GangreNosum and Hidradenitis Suppurativa. Sun NZ, Ro T, Jolly P, Sayed CJ. J Clin Aesthet Dermatol. 2017 Sep;10(9):36-38.
21	The expanding spectrum of clinical phenotypes associated with PSTPIP1 mutations: from PAPA to PAMI syndrome and beyond. Klötgen HW, Beltraminelli H, Yawalkar N, van Gijn ME, Holzinger D, Borradori L. Br J Dermatol. 2018 Apr;178(4):982-983.

Article ID	Article
22	Imaging findings of sterile pyogenic arthritis, pyoderma gangrenosum and acne (PAPA) syndrome: differential diagNosis and review of the literature. Martinez-Rios C, Jariwala MP, Highmore K, Duffy KW, Spiegel L, Laxer RM, Stimec J. Pediatr Radiol. 2019 Jan;49(1):23-36.
23	Burlakov V, Kozlova A, Mersiyanova I, et alTHU0638 Clinical characteristic of a group of patients with pstpip1-associated myeloid-related proteinemia inflammatory syndrome (PAMI)Annals of the Rheumatic Diseases 2018;77:514.
24	PAPASH, PsAPASH and PASS autoinflammatory syndromes: phenotypic heterogeneity, common biological signature and respons[illegible] to immunosuppressive regimens. Gottlieb J, Madrange M, Gardair C, Sbidian E, Frazier A, Wolkenstein P, Hickman G, Schneider P, Baudry C, Claudepierre P, Bertheau P, Richette P, Smahi A, Bachelez H. Br J Dermatol. 2019 Oct;181(4):866-869.
25	Clinical picture of 7 PAPA patients followed in a single pediatric rheumatologic center. Federici, Silvia & Celani, Camilla & Messia, Virginia & Marucci, Giulia & Kessel, Christoph & De Benedetti, Fabrizio & Insalaco, Antonella. (2019). Annals of the Rheumatic Diseases. 78. 1337.2-1338. 10.1136/annrheumdis-2019-eular.6176.
26	PAPA syndrome: novelties from the Eurofever registry. Roberta Caorsi, D.M. Pediatric Rheumatology online Journal, 17(S1), (2019).
27	Tildrakizumab in the treatment of PASH syndrome: A potential novel therapeutic target. Kok Y, Nicolopoulos J, Varigos G, Howar[illegible] A, Dolianitis C. Australas J Dermatol. 2020 Aug;61(3):e373-e374.
28	PASS: a rare syndrome within the autoinflammatory diseases that still lacks a genetic marker. Schwob E, Bessis D, Boursier G, Kottler D, Guillot B, Lerisson M, Girard C. J Eur Acad Dermatol Venereol. 2020 Sep;34(9):e478-e480.
29	Brief report: geNotype, pheNotype, and clinical course in five patients with PAPA syndrome (pyogenic sterile arthritis, pyoderma gangreNosum, and acne). Demidowich AP, Freeman AF, Kuhns DB, Aksentijevich I, Gallin JI, Turner ML, Kastner DL, Holland SM. Arthritis Rheum. 2012 Jun;64(6):2022-7.
30	Association of pyoderma gangrenosum, acne, and suppurative hidradenitis (PASH) shares genetic and cytokine profiles with other autoinflammatory diseases. Marzano AV, Ceccherini I, Gattorno M, Fanoni D, Caroli F, Rusmini M, Grossi A, De Simone C, Borghi OM, Meroni PL, Crosti C, Cugno M. Medicine (Baltimore). 2014 Dec;93(27):e187.
31	HSCT is effective in patients with PSTPIP1-associated myeloid-related proteinemia inflammatory (PAMI) syndrome. Laberko A, Burlakov V, Maier S, Abinun M, Skinner R, Kozlova A, Suri D, Lehmberg K, Müller I, Balashov D, Novichkova G, Holzinger D, Gennery AR, Shcherbina A. J Allergy Clin Immunol. 2021 Jul;148(1):250-255.e1.
32	Haematological involvement associated with a mild autoinflammatory phenotype, in two patients carrying the E250K mutation of PSTPIP1. Belelli E, Passarelli C, Pardeo M, Holzinger D, De Benedetti F, Insalaco A. Clin Exp Rheumatol. 2017 Nov-Dec;35 Suppl 108(6):113-115.

Article ID	Article
33	Immunological repertoire linked to PSTPIP1-associated myeloid-related inflammatory (PAMI) syndrome. Mendonça LO, Terreri MT, Osaku FM, Barros SF, Köhler KF, Prado AI, Barros MT, Kalil J, Castro FFM. Pediatr Rheumatol Online J. 2021 Aug 16;19(1):126.
34	Kidney Involvement in PSTPIP1 Associated Inflammatory Diseases (PAID): A Case Report and Review of the Literature. Borgia P, Papa R, D'Alessandro M, Caorsi R, Piaggio G, Angeletti A, Ceccherini I, Ghiggeri GM, Gattorno M. Front Med (Lausanne). 2021 Oct 29;8:759092.
35	Hemolysis and Neurologic Impairment in PAMI Syndrome: novel Characteristics of an Elusive Disease. Del Borrello G, Guardo D [illegible] Micalizzi C, Ceccherini I, Miano M, Gattorno M, Dufour C. Pediatrics. 2021 Mar;147(3):e20200784.
36	Successful combined antibiotic therapy with oral clindamycin and oral rifampicin for pyoderma gangreNosum in patient with PASH syndrome. Lamiaux M, Dabouz F, Wantz M, Lebas D, Lasek A, Courivaud D, ModiaNo P. JAAD Case Rep. 2017 Dec 18;4(1):17-21.

Table S3. List of excluded studies with reasons for exclusion.

ID	Article	Reason for exclusion
1.	Anakinra therapy in a patient with multifocal pyoderma gangrenosum and hidradenitis suppurativa. Oymanns M, Kreuter A, Assaf C. Hautarzt. 2021 May;72(5):435-438.	No English
2.	Rare cases of PAMI syndrome in both father and son with the same missense mutation in PSTPIP1 gene and literature review. Huang X, Xu M, Dai S, Wang M, Zheng H, Zeng K, Li L. J Dermatol. 2021 Apr;48(4):519-528.	No anti-IL-1 treament.
3.	Pyoderma gangrenosum and concomitant hidradenitis suppurativa--rapid response to canakinumab (anti-IL-1β). Jaeger T, Andres C, Grosber M, Zirbs M, Hein R, Ring J, Traidl-Hoffmann C. Eur J Dermatol. 2013 May-Jun;23(3):408-10.	No PAID syndrome
4.	Autoinflammatory syndromes associated with hidradenitis suppurativa and/or acne. Vinkel C, Thomsen SF. Int J Dermatol. 2017 Aug;56(8):811-818.	Review
5.	A Snapshot on the On-Label and Off-Label Use of the Interleukin-1 Inhibitors in Italy among Rheumatologists and Pediatric Rheumatologists: A Nationwide Multi-Center Retrospective Observational Study. Vitale A, Insalaco A, Sfriso P, Lopalco G, Emmi G, Cattalini M, Manna R, Cimaz R, Priori R, Talarico R, Gentileschi S, de Marchi G, Frassi M, Gallizzi R, Soriano A, Alessio M, Cammelli D, Maggio MC, Marcolongo R, La Torre F, Fabiani C, Colafrancesco S, Ricci F, Galozzi P, Viapiana O, Verrecchia E, Pardeo M, Cerrito L, Cavallaro E, Olivieri AN, Paolazzi G, Vitiello G, Maier A, Silvestri E, Stagnaro C, Valesini G, Mosca M, de Vita S, Tincani A, Lapadula G, Frediani B, De Benedetti F, Iannone F, Punzi L, Salvarani C, Galeazzi M, Rigante D, Cantarini L. Front Pharmacol. 2016 Oct 24;7:380.	No PAID syndrome. Review.
6.	New described dermatological disorders. Gönül M, Cevirgen Cemil B, Keseroglu HO, Kaya Akis H. Biomed Res Int. 2014;2014:616973.	Review
7.	Phenotype variability of autoinflammatory disorders in the pediatric patient: A pictorial overview. Rigante D. Journal of Evidence-Based Medicine (2020) 13:3 (227-245).	Review
8.	Investigational drugs in clinical trials for Hidradenitis Suppurativa. Theut Riis P. Et al. Expert Opinion on Investigational Drugs (2018) 27:1 (43-53).	No PAID syndrome
9.	Endpoints of clinical trials for Hidradenitis Suppurativa: Proceedings of a round-table session. Zouboulis C.C., Gulliver W., Ingram J., Kirby B., Giamarellos-Bourboulis	No PAID syndrome

	E.J., Podda M., Tzellos T., Jemec G.B.E. Experimental Dermatology (2020) 29:S1 (67-72).	
10.	Clinical Disease Patterns in a Regional Swiss Cohort of 34 Pyoderma Gangrenosum Patients. Kolios A.G.A. Et al. Dermatology (2018) 233:4 (268-276).	No PAID syndrome
11.	Targeted treatments for hidradenitis suppurativa: a review of the current literature and ongoing clinical trials. Maarouf M. Et al. Journal of Dermatological Treatment (2017) (1-9).	No PAID syndrome
12.	Canakinumab for severe hidradenitis suppurativa: Preliminary experience in 2 cases. Houriet C. Et al. JAMA Dermatology (2017) 153:11 (1195-1197).	No PAID syndrome
13.	Drug management of neutrophilic dermatoses. Garcovich S. Et al. Expert Review of Clinical Pharmacology (2017) 10:10 (1119-1128).	No PAID syndrome
14.	Pyoderma Gangrenosum: A Current Problem as Much as an Unknown One. Vallini V. Et al. International Journal of Lower Extremity Wounds (2017) 16:3 (191-201).	No PAID syndrome
15.	SAPHO and Recurrent Multifocal Osteomyelitis. Greenwood S. Et al. Radiologic Clinics of North America (2017) 55:5 (1035-1053).	No PAID syndrome
16.	Hidradenitis suppurativa unresponsive to canakinumab treatment: A case report. Tekin B. Et al. Indian Journal of Dermatology, Venereology and Leprology (2017) 83:5 (615-617).	No PAID syndrome
17.	Evolving frontiers in the treatment of periodic fever, aphthous stomatitis, pharyngitis, cervical adenitis (PFAPA) syndrome. Rigante D. Et al. Israel Medical Association Journal (2017) 19:7 (444-447).	No PAID syndrome
18.	PAPA, PASH and PAPASH Syndromes: Pathophysiology, Presentation and Treatment. Cugno M. Et al. American Journal of Clinical Dermatology (2017) 18:4 (555-562).	Review
19.	Canakinumab efficacy in refractory adult-onset PFAPA syndrome. Lopalco G. Et al. International Journal of Rheumatic Diseases (2017) 20:8 (1050-1051).	No PAID syndrome
20.	Use of biological treatments in patients with hidradenitis suppurativa. Martin-Ezquerra G. Et al. Giornale Italiano di Dermatologia e Venereologia (2017) 152:4 (373-378).	No PAID syndrome
21.	Medical management of hidradenitis suppurativa. Orenstein L.A.V. Et al. Seminars in Cutaneous Medicine and Surgery (2017) 36:2 (62-66).	No PAID syndrome
22.	Physicians perspectives on the diagnosis and management of periodic fever, aphthous stomatitis, pharyngitis, and cervical adenitis (PFAPA) syndrome. Manthiram K. Et al. Rheumatology International (2017) 37:6 (883-889).	No PAID syndrome

23.	Potential of IL-1, IL-18 and inflammasome inhibition for the treatment of inflammatory skin diseases. Fenini G. Et al. Frontiers in Pharmacology (2017) 8:MAY Article Number: 278.	Review
24.	Biologics in patients with skin diseases. Veilleux M.S. Et al. Journal of Allergy and Clinical Immunology (2017) 139:5 (1423-1430).	Review
25.	Biologic Therapy in the Treatment of Chronic Skin Disorders. Fernandez J.M. Et al. Immunology and Allergy Clinics of North America (2017) 37:2 (315-327).	Review
26.	Pediatric pyoderma gangrenosum: a systematic review and update. Kechichian E. Et al. International Journal of Dermatology (2017) 56:5 (486-495).	Review
27.	Interventions for hidradenitis suppurativa updated summary of an original cochrane review. Ingram J.R. JAMA Dermatology (2017) 153:5 (458-459).	Review
28.	Hidradenitis suppurativa: From pathogenesis to diagnosis and treatment. Napolitano M. Et al. Clinical, Cosmetic and Investigational Dermatology (2017) 10 (105-115).	No PAID syndrome
29.	Successful Treatment of Autoimmune Disease-Associated Pyoderma Gangrenosum with the IL-1 Receptor Antagonist Anakinra: A Case Series of 3 Patients. Beynon C. Et al. Journal of Clinical Rheumatology (2017) 23:3 (181-183).	No PAID syndrome
30.	Current systemic treatment strategies for hidradenitis suppurativa. Ponikowska M. Et al. Expert Opinion on Orphan Drugs (2017) 5:3 (241-251).	Review
31.	Association of hidradenitis suppurativa and familial Mediterranean fever: A case series of 6 patients. Abbara S. Et al. Joint Bone Spine (2017) 84:2 (159-162).	No PAID syndrome
32.	Familial mediterranean fever treated with anakinra: A case report. Espaldora-Hernández J. Et al. Reumatologia Clinica (2017) 13:2 (120-121).	No PAID syndrome
33.	Treatments for hidradenitis suppurativa. Andersen R.K. Et al. Clinics in Dermatology (2017) 35:2 (218-224).	Review
34.	High level of serum human interleukin-18 in a patient with pyogenic arthritis, pyoderma gangrenosum and acne syndrome. Kanameishi S. Et al. Journal of the European Academy of Dermatology and Venereology (2017) 31:2 (e115-e116).	No anti-IL-1 treatment
35.	Severe hidradenitis suppurativa responding to treatment with secukinumab. Thorlacius L. Et al. Experimental Dermatology (2017) 26 Supplement 1 (3).	No PAID syndrome
36.	Pharmacokinetics and pharmacodynamics of canakinumab in patients with periodic fever syndromes (colchicine-resistant FMF, HIDS/MKD and TRAPS): Results from a phase III pivotal umbrella trial. De Benedetti F. Et al. Pediatric Rheumatology (2017) 15 Supplement 1.	No PAID syndrome
37.	Efficacy and safety of Canakinumab in patients with periodic fever syndromes (col-	No PAID syndrome

	chicine-resistant fmf, hids/mkd and traps): Results from a phase 3, pivotal, umbrella trial. De Benedetti F. ET AL. Pediatric Rheumatology (2017) 15 Supplement 1.	
38.	Effect of canakinumab on health-related quality of life in patients with periodic fever syndromes. Lachmann H. Et al. Pediatric Rheumatology (2017) 15 Supplement 1.	No PAID syndrome
39.	Other autoinflammatory disease genes in an FMF-prevalent population: A homozygous MVK mutation and a heterozygous TNFRSF1A mutation in two different Turkish families with clinical FMF. Karacan I. Et al. Clinical and Experimental Rheumatology (2017) 35 Supplement108 (S75-S81).	No PAID syndrome
40.	Acne fulminans. Dall'Oglio F., Puglisi D.F., Nasca M.R., Micali G. Giornale italiano di dermatologia e venereologia : organo ufficiale, Societa italiana di dermatologia e sifilografia (2020).	Review
41.	Introduction-Biologics in Dermatology: Indications and Off-Label Usage. Puig L. Current Problems in Dermatology (Switzerland) (2017) 53 (vii-x).	Review
42.	Pediatric Pyoderma Gangrenosum: A Retrospective Review of Clinical Features, Etiologic Associations, and Treatment. Schoch J.J. Et al. Pediatric Dermatology (2017) 34:1 (39-45).	Review
43.	Treatment options for pyoderma gangrenosum. Quist S.R. Et al. JDDG - Journal of the German Society of Dermatology (2017) 15:1 (34-40).	Review
44.	The Role of Interleukin-1 in Inflammatory and Malignant Human Skin Diseases and the Rationale for Targeting Interleukin-1 Alpha. Bou-Dargham M.J. Et al. Medicinal Research Reviews (2017) 37:1 (180-216).	Review
45.	Pyrin-associated autoinflammation with neutrophilic dermatosis (PAAND): Preliminary results of a proof-of-concept trial with anakinra. Eyck L.V. Et al. Pediatric Rheumatology (2017) 15 Supplement 1.	No PAID syndrome
46.	Hidradenitis suppurativa: An update on connecting the tracts. Hamzavi I.H. Et al. F1000Research (2017) 6 Article Number: 1272.	Review
47.	Review of Current Immunologic Therapies for Hidradenitis Suppurativa. Shanmugam V.K. Et al. International Journal of Rheumatology (2017) 2017 Article Number: 8018192.	Review
48.	Unexplained intermittent fever: PFAPA. Simmons A. Et al. Paediatrics and Child Health (United Kingdom) (2016) 26:12 (559-560).	No PAID syndrome
49.	Pharmacokinetic and pharmacodynamic characteristics of canakinumab in patients with periodic fever syndromes: Results from a phase iii pivotal umbrella trial. Frenkel J. ET AL. Arthritis and Rheumatology (2016) 68 Supplement 10 (3103-3104).	No PAID syndrome

50.	Effect of canakinumab treatment on health-related quality of life in patients with periodic fever syndromes. Simon A. ET AL. Arthritis and Rheumatology (2016) 68 Supplement 10 (3104-3106).	No PAID syndrome
51.	The role of interleukin-1 in pyoderma gangrenosum. Galimberti R.L. Et al. JAAD Case Reports (2016) 2:5 (366-368).	No PAID syndrome
52.	Cerebral vascular findings in PAPA syndrome: Cerebral arterial vasculopathy or vasculitis and a posterior cerebral artery dissecting aneurysm. Khatibi K. Et al. Journal of NeuroInterventional Surgery (2016) 8:8 (e29).	No anti-IL-1 treatment
53.	New treatment strategies for hidradenitis suppurativa. Andersen R. Et al. Drugs of Today (2016) 52:8 (439-451).	Review
54.	Synovitis, acne, pustulosis, hyperostosis, and osteitis (SAPHO) syndrome "A challenging diagnosis not to be missed. Zimmermann P. Et al. Journal of Infection (2016) 72 Supplement (S106-S114).	No PAID syndrome
55.	Periodic fever, aphthous stomatitis, pharyngitis, and adenitis (PFAPA) syndrome. Ali N.S. Et al. Clinics in Dermatology (2016) 34:4 (482-486).	No PAID syndrome
56.	PFAPA syndrome: A review on treatment and outcome. Vanoni F. Et al. Pediatric Rheumatology (2016) 14:1 Article Number: 38.	No PAID syndrome
57.	A phase III pivotal umbrella trial of canakinumab in patients with autoinflammatory periodic fever syndromes (colchicine resistant FMF, HIDS/MKD and TRAPS). De Benedetti F. ET AL. Annals of the Rheumatic Diseases (2016) 75 Supplement 2 (615-616).	No PAID syndrome
58.	Pharmacokinetics and pharmacodynamics of canakinumab in patients with autoinflammatory periodic fever syndromes (colchicine resistant FMF, HIDS/MKD and traps). De Benedetti F. ET AL. Annals of the Rheumatic Diseases (2016) 75 Supplement 2 (397-398).	No PAID syndrome
59.	Canakinumab improves patient reported outcomes in patients with periodic fever syndromes. Lachmann H. Et al. Annals of the Rheumatic Diseases (2016) 75 Supplement 2 (616).	No PAID syndrome
60.	SAPHO Syndrome: Current Developments and Approaches to Clinical Treatment. Firinu D. Et al. Current Rheumatology Reports (2016) 18:6 Article Number: 35.	No PAID syndrome
61.	Failure of anakinra in a CASE of severe Hidradenitis suppurativa. Russo V. Et al. Journal of Drugs in Dermatology (2016) 15:6 (772-774).	No PAID syndrome
62.	Periodic Fever, Aphthous Stomatitis, Pharyngitis, and Cervical Adenitis (PFAPA) Syndrome: a Review of the Pathogenesis. Theodoropoulou K. Et al. Current Rheuma-	No PAID syndrome

	tology Reports (2016) 18:4 Article Number: 18.	
63.	Neutrophilic panniculitis associated with alpha-1-antitrypsin deficiency: An update. Blanco I. Et al. British Journal of Dermatology (2016) 174:4 (753-762).	Review
64.	Approach to and Management of the Neutrophilic Dermatoses. Kurtzman D. Et al. Current Dermatology Reports (2016) 5:1 (18-29).	Review
65.	An Update on Medical Treatment Options for Hidradenitis Suppurativa. Deckers I.E. Et al. Drugs (2016) 76:2 (215-229).	Review
66.	Pyoderma gangraenosum -from the right diagnostic investigations to targeted therapy. JockenhÌfer F. Et al. Phlebologie (2016) 45:5 (333-340).	Review
67.	Demographic, clinical and therapeutic findings in a monocentric cohort of adult patients with suspected PFAPA syndrome. Vitale A. Et al. Clinical and Experimental Rheumatology (2016) 34 Supplement102 (77-81).	No PAID syndrome
68.	Safety and efficacy of anakinra in severe hidradenitis suppurativa a randomized clinical trial. Tzanetakou V. Et al. JAMA Dermatology (2016) 152:1 (52-59).	No PAID syndrome
69.	Randomized Controlled Trials for the Treatment of Hidradenitis Suppurativa. Van Rappard D.C. Et al. Dermatologic Clinics (2016) 34:1 (69-80).	No PAID syndrome
70.	Guidelines for the management and treatment of periodic fever syndromes: Periodic fever, aphthous stomatitis, pharyngitis and adenitis syndrome. Terreri M.T.R.A. Et al. Revista Brasileira de Reumatologia (2016) 56:1 (52-57).	No PAID syndrome
71.	Refractory peristomal pyoderma gangrenosum successfully treated with intravenous immunoglobulin: A case report. McAllister B.P. Et al. American Journal of Gastroenterology (2015) 110:12 (1739-1740).	No PAID syndrome
72.	Towards a targeted therapy for pyoderma gangrenosum. Costanzo A et al. British Journal of Dermatology (2015) 173:5 (1124).	No PAID syndrome
73.	Treatment of hidradenitis suppurativa with biologic medications. Lee R.A. Et al. Journal of the American Academy of Dermatology (2015) 73:5 Supplement 1 (S82-S88).	No PAID syndrome
74.	Canakinumab in adults with steroid-refractory pyoderma gangrenosum. Kolios A.G.A. Et al. British Journal of Dermatology (2015) 173:5 (1216-1223).	No PAID syndrome
75.	Interventions for hidradenitis suppurativa. Ingram J.R. Et al. Cochrane Database of Systematic Reviews (2015) 2017:2 Article Number: CD010081.	Review
76.	Anti-IL1-monoclonal antibody in two adult patients with PFAPA Syndrome-a single centre experience. Kortus-GÌtze B. Et al. Pediatric Rheumatology (2015) 13 SUPPL. 1 (211DUMMY).	No PAID syndrome

77.	Adult PFAPA-a single centre experience. Donnelly O. Et al. Pediatric Rheumatology (2015) 13 SUPPL. 1 (252DUMMY).	No PAID syndrome
78.	Long-term efficacy of IL-1 blockers in PAPA patients. Finetti M. Et al. Pediatric Rheumatology (2015) 13:1 Article Number: P207.	Review
79.	Hidradenitis suppurativa and familial Mediterranean fever: A report of 6 cases and literature review. Abbara S. Rt al. Pediatric Rheumatology (2015) 13 SUPPL. 1 (181DUMMY).	No PAID syndrome
80.	Biologics for SAPHO syndrome: A single centre experience. Murgia G. ET AL. Allergy: European Journal of Allergy and Clinical Immunology (2015) 70 SUPPL. 101 (418-419).	No PAID syndrome
81.	Successful treatment of pyoderma gangrenosum with the interleukin-1 receptor antagonist anakinra: A case series of three patients. Chin M.F. Et al. British Journal of Dermatology (2015) 173 SUPPL. 1 (67-68).	No PAID syndrome
82.	Pyoderma gangrenosum: Challenges and solutions. Gameiro A. Et al. Clinical, Cosmetic and Investigational Dermatology (2015) 8 (285-293).	Review
83.	Neutrophilic dermatosis: Disease mechanism and treatment. Maalouf D. Et al. Current Opinion in Hematology (2015) 22:1 (23-29).	Review
84.	The Medical and Laser Management of Hidradenitis Suppurativa. Hughes R. Et al. American Journal of Clinical Dermatology (2015) 16:2 (111-123).	Review
85.	Therapy of pyoderma gangrenosum in Germany: Results of a survey among wound experts. Al Ghazal P et al. JDDG - Journal of the German Society of Dermatology (2015) 13:4 (317-325).	Review
86.	Effective strategies for the management of pyoderma gangrenosum: A comprehensive review. Patel F. Et al. Acta Dermato-Venereologica (2015) 95:5 (525-531).	Review
87.	Anakinra improves pyoderma gangrenosum in psoriatic arthritis: A case report. Acquitter M. Et al. Annals of Internal Medicine (2015) 163:1 (70-71).	No PAID syndrome
88.	Refractory pyoderma gangrenosum in an infant. Etzler C. Et al. Pediatric Dermatology (2015) 32:1 (156-157).	No PAID syndrome
89.	Canakinumab for the treatment of different refractory autoinflammatory disorders. Grein I.H.R. Et al. Pediatric Rheumatology (2014) 12 SUPPL. 1.	No PAID syndrome
90.	Treatment with anti IL-1 and infectious complications in paediatric patients with systemic juvenile idiophatic arthritis and autoinflammatoy diseases. Fernandez-Silveira L. Et al. Annals of the Rheumatic Diseases (2014) 73 SUPPL. 2.	No PAID syndrome
91.	An open-label study of anakinra for the treatment of moderate to severe hidradenitis	No PAID syndrome

	suppurativa. Leslie K.S. Et al. Journal of the American Academy of Dermatology (2014) 70:2 (243-251).	
92.	Biological treatments for SAPHO syndrome: An update. Firinu D. Et al. Inflammation and Allergy - Drug Targets (2014) 13:3 (199-205).	No PAID syndrome
93.	Pyoderma gangrenosum: Pathogenetic oriented treatment approaches. Wollina U. Et al. Wiener Medizinische Wochenschrift (2014) 164:13-14 (263-273).	No PAID syndrome
94.	Certolizumab for the treatment of refractory disseminated pyoderma gangrenosum associated with rheumatoid arthritis. Cinotti E. Et al. Clinical and Experimental Dermatology (2014) 39:6 (750-751).	No PAID syndrome
95.	The efficacy of anti- IL-1 treatment in three patients with coexisting familial Mediterranean fever and multiple sclerosis. Ozdogan H., Ugurlu S., Uygunoglu U., Tutuncu M., Gul A., Akman G., Siva A. Multiple Sclerosis and Related Disorders (2020) 45 Article Number: 102332.	No PAID syndrome
96.	Intravenous anakinra for cytokine storm syndromes. Martis N., Audemard-Verger A. The Lancet Rheumatology (2020) 2:9 (e522).	Review
97.	Long-term management of patients with PFAPA syndrome. Yıldız E., Kuzu S., Kahveci O.K., Ulu Ş., Bucak A. European Archives of Oto-Rhino-Laryngology (2020) 277:8 (2335-2339).	No PAID syndrome
98.	IL-17 in inflammatory skin diseases psoriasis and hidradenitis suppurativa. Fletcher J.M., Moran B., Petrasca A., Smith C.M. Clinical and Experimental Immunology (2020) 201:2 (121-134).	Review
99.	Successful treatment of severe hidradenitis suppurativa with anakinra. Zarchi K. Et al. JAMA Dermatology (2013) 149:10 (1192-1194).	No PAID syndrome
100	A case report of a patient with crohn's disease complicated by pyoderma gangrenosum treated with intravenous immunoglobulin. Yoo L. Et al. American Journal of Gastroenterology (2013) 108 SUPPL. 1 (S421).	No PAID syndrome
101	Trends of SAPHO therapy: Should we be content? Rozin A.P. Et al. Current Rheumatology Reviews (2013) 9:1 (28-33).	No PAID syndrome
102	Failure of anti-interleukin-1 therapy in severe hidradenitis suppurativa: A case report. Van Der Zee H.H et al. Dermatology (2013) 226:2 (97-100).	No PAID syndrome
103	Periodic fever syndromes in the Czech Republic: A single-centre experience. Krakovsk V. Et al. Annals of the Rheumatic Disease (2013) 71 SUPPL. 3.	No PAID syndrome
104	SAPHO Syndrome. Carneiro S. Et al. Rheumatic Disease Clinics of North America (2013) 39:2 (401-418).	No PAID syndrome

105	Therapeutic strategies for SAPHO syndrome. Trotta F. Et al. Expert Opinion on Orphan Drugs (2013) 1:10 (773-780).	No PAID syndrome
106	The SAPHO Syndrome. Nguyen M.T. Et al. Seminars in Arthritis and Rheumatism (2012) 42:3 (254-265).	No PAID syndrome
107	Hidradenitis suppurativa: Viewpoint on clinical phenotyping, pathogenesis and novel treatments. Van der Zee H.H. Et al. Experimental Dermatology (2012) 21:10 (735-739).	Review
108	An overview of interleukin-1 receptor Antagonist, Anakinra, in the treatment of cutaneous diseases. Pazyar N. Et l. Current Clinical Pharmacology (2012) 7:4 (271-275).	Review
109	IBD-related pyoderma gangrenosum in the biologic era: A single centre, 5-year case series and systematic literature review. Agarwal A. Et al. Journal of Gastroenterology and Hepatology (2012) 27 SUPPL. 4 (96).	Review
110	A case of resistant adult-onset periodic fever, aphtous stomatitis, pharyngitis and cervical adenitis (PFAPA) syndrome responsive to anakinra. Cantarini L. Et al. Clinical and Experimental Rheumatology (2012) 30:4 (593).	No PAID syndrome
111	Indications and modes of use for interleukin (IL)-1 antagonists in inflammatory dermatosis: A new therapeutic approach to immune-mediated inflammatory diseases. Lipsker D. Et al. Annales de Dermatologie et de Venereologie (2012) 139:6-7 (459-467).	Review
112	Anakinra treatment of SAPHO syndrome: Short-term results of an open study. Wendling D. Et al. Annals of the Rheumatic Diseases (2012) 71:6 (1098-1100).	No PAID syndrome
113	Interleukin-1, inflammasomes, autoinflammation and the skin. Contassot E. Et al. Swiss Medical Weekly (2012) 142:MAY Article Number: w13590.	Review
114	Failure of anakinra treatment of pyoderma gangrenosum in an IBD patient and relevance to the PSTPIP1 gene. Lin Z. ET AL. Inflammatory Bowel Diseases (2011) 17:6 (E41-E42).	No PAID syndrome
115	Periodic fever, aphthous stomatitis, pharyngitis, and adenitis (PFAPA) is a disorder of innate immunity and Th1 activation responsive to IL-1 blockade. Stojanov S. Et al. Proceedings of the National Academy of Sciences of the United States of America (2011) 108:17 (7148-7153).	No PAID syndrome
116	Pyoderma gangraenosum. Wollina U. Et al. Current Opinion in Rheumatology (2011) 23:1 (50-56).	Review
117	Targeting the IL-1 family members in skin inflammation. Jensen L.E. Current Opinion in Investigational Drugs (2010) 11:11 (1211-1220).	Review

118	Hidradenitis suppurativa and concomitant pyoderma gangrenosum: A case series and literature review. Hsiao J.L. Et al. Archives of Dermatology (2010) 146:11 (1265-1270).	No anti-IL-1 treatment
119	Biologics in dermatologic therapy - An update. Coondoo A. Indian Journal of Dermatology (2009) 54:3 (211-220).	Review
120	The rapidly evolving role of anakinra in dermatology: A double-edged sword. Kapoor S. Journal of the American Academy of Dermatology (2008) 59:6 (1092-1093).	Review
121	Cytokine therapy in dermatology. Asadullah K. Et al. Experimental Dermatology (2002) 11:2 (97-106).	Review
122	Immunopathogenesis of hidradenitis suppurativa and response to anti-TNF-a therapy. Lowe M.M. Et al. JCI Insight (2020) 5:19 Article Number: e139932.	No PAID syndrome
123	Pyoderma gangrenosum: From historical perspectives to emerging investigations. Hobbs M.M., Ortega-Loayza A.G. International Wound Journal (2020) 17:5 (1255-1265).	No PAID syndrome
124	Hidradenitis suppurativa: New drugs, old challenges. Elston D.M. Journal of the American Academy of Dermatology (2020) 83:3 (733-734).	No PAID syndrome
125	Intravenous anakinra for cytokine storm syndromes. Martis N., Audemard-Verger A. The Lancet Rheumatology (2020) 2:9 (e522).	No PAID syndrome
126	Medical and Surgical Management of Hidradenitis Suppurativa: A Review of International Treatment Guidelines and Implementation in General Dermatology Practice. Orenstein L.A.V., Nguyen T.V., Damiani G., Sayed C., Jemec G.B.E., Hamzavi I. Dermatology (2020) 236:5 (393-412).	No PAID syndrome
127	Emerging medical treatments for hidradenitis suppurativa. Lyons A.B., Shabeeb N., Nicholson C.L., Braunberger T.L., Peacock A., Hamzavi I.H. Journal of the American Academy of Dermatology (2020) 83:2 (554-562).	No PAID syndrome
128	Primary imputation methods impact efficacy results in hidradenitis suppurativa clinical trials. Frew J.W. Journal of the American Academy of Dermatology (2020) 83:2 (663-665).	No PAID syndrome
129	Efficacy and Safety of Canakinumab in sJIA Patients with and without Fever at Baseline: Results from an Open-label, Active Treatment Extension Study. Brunner H.I. Et al. Arthritis & rheumatology (Hoboken, N.J.) (2020).	No PAID syndrome
130	Hidradenitis suppurativa management using tumor necrosis factor inhibitors in patients younger than 18 years: A series of 12 cases. Fougerousse A.-C., Reguiai Z., Roussel A., Bécherel P.-A. Journal of the American Academy of Dermatology (2020)	No PAID syndrome

	83:1 (199-201).	
131	Conversion to positive latent tuberculosis infection status is low in patients with hidradenitis suppurativa taking biologic medications. Ellis A., Khanna U., Galadari A., Fernandez A.P. Journal of the American Academy of Dermatology (2020) 83:1 (246-248).	No PAID syndrome
132	856 Arthritis-associated pyoderma gangrenosum: A systematic review. Sawka E., Friedman M., Ortega-Loayza A. Journal of Investigative Dermatology (2020) 140:7 Supplement (S111).	No PAID syndrome
133	Current Therapeutic Options for the Main Monogenic Autoinflammatory Diseases and PFAPA Syndrome: Evidence-Based Approach and Proposal of a Practical Guide. Soriano A. Et al. Frontiers in Immunology (2020) 11 Article Number: 865.	No PAID syndrome
134	Biologic therapies for the treatment of hidradenitis suppurativa. Rosales Santillan M., Morss P.C., Porter M.L., Kimball A.B. Expert Opinion on Biological Therapy (2020) 20:6 (621-633).	No PAID syndrome
135	Efficacy of anakinra treatment in pediatric rheumatic diseases; a single-center experience. Demir F., Gurler E., Colak B., Sozeri B. Annals of the Rheumatic Diseases (2020) 79:SUPPL 1 (1783-1784).	No PAID syndrome
136	Hidradenitis Suppurativa as a Potential Subtype of Autoinflammatory Keratinization Disease. Nomura T. Frontiers in Immunology (2020) 11 Article Number: 847.	No PAID syndrome
137	Hidradenitis suppurativa encounters in a national electronic health record database notable for low dermatology utilization, infrequent biologic prescriptions, and frequent opiate prescriptions. Wehner M.R., Micheletti R., Noe M.H., Linos E., Margolis D.J., Naik H.B. Journal of the American Academy of Dermatology (2020) 82:5 (1239-1241).	No PAID syndrome
138	Hidradenitis suppurativa: Current and emerging treatments. Goldburg S.R., Strober B.E., Payette M.J. Journal of the American Academy of Dermatology (2020) 82:5 (1061-1082).	No PAID syndrome
139	Acne fulminans and Cutibacterium acnes phylotypes. Bocquet-Trémoureux S. Et al. Journal of the European Academy of Dermatology and Venereology (2020) 34:4 (827-833).	No PAID syndrome
140	A concise clinician's guide to therapy for hidradenitis suppurativa. Nesbitt E., Clements S., Driscoll M. International Journal of Women's Dermatology (2020) 6:2 (80-84).	No PAID syndrome
141	Diagnosis and novel clinical treatment strategies for pyoderma gangrenosum. Goldust	No PAID syndrome

	M. Et al. Expert Review of Clinical Pharmacology (2020) 13:2 (157-161).	
142	Evaluating patients' unmet needs in hidradenitis suppurativa: Results from the Global Survey Of Impact and Healthcare Needs (VOICE) Project. Garg A. Et al. Journal of the American Academy of Dermatology (2020) 82:2 (366-376).	No PAID syndrome
143	Hidradenitis suppurativa: What's new in pathogenesis and management. Wark K.J.L., Cains G.D. Medicine Today (2020) 21:2 (19-23).	No PAID syndrome
144	Ustekinumab with intravenous infusion: Results in hidradenitis suppurativa. Romaní J., Vilarrasa E., Martorell A., Fuertes I., Ciudad C., Molina-Leyva A. Dermatology (2020) 236:1 (21-24).	No PAID syndrome
145	Off-Label studies on anakinra in dermatology: a review. Tegtmeyer K., Atassi G., Zhao J., Maloney N.J., Lio P.A. Journal of Dermatological Treatment (2020).	Review
146	MABp1 targeting interleukin-1α in hidradenitis suppurativa ineligible for adalimumab treatment: results of the open-label extension period. Kanni T., Argyropoulou M., Dinarello C.A., Simard J., Giamarellos-Bourboulis E.J. Clinical and Experimental Dermatology (2020).	No PAID syndrome
147	The off-label use of anakinra in pediatric systemic autoinflammatory diseases. Maniscalco V., Abu-Rumeileh S., Mastrolia M.V., Marrani E., Maccora I., Pagnini I., Simonini G. Therapeutic Advances in Musculoskeletal Disease (2020) 12.	No PAID syndrome
148	The most recent advances in understanding and managing hidradenitis suppurativa. Hamzavi I.H., Narla S., Lyons A.B. F1000Research (2020) 9 Article Number: 1049.	No PAID syndrome
149	Hidradenitis suppurativa: Rapid evidence review. Wipperman J., Bragg D.A., Litzner B. American Family Physician (2019) 100:9 (562-569).	Review
150	Long-term therapy with anakinra in hidradenitis suppurativa in three patients. André R., Marescassier H., Gabay C., Pittet B., Laffitte E. International Journal of Dermatology (2019) 58:11 (e208-e209).	No PAID syndrome
151	Tuscan consensus on the diagnosis and treatment of hidradenitis suppurativa. Prignano F., Pescitelli L., Giani I., Dini V., Romanelli M. Journal of the European Academy of Dermatology and Venereology (2019) 33:S6 (21-24).	No PAID syndrome
152	Biologic Treatment for Hidradenitis Suppurativa. Flood K.S., Porter M.L., Kimball A.B. American Journal of Clinical Dermatology (2019) 20:5 (625-638).	No PAID syndrome
153	Effectiveness and safety of off-label use of tocilizumab in refractory autoimmune diseases: A multicenter study. Michaud M. Arthritis and Rheumatology (2019) 71 Supplement 10 (2190-2191).	No PAID syndrome
154	349 Hidradenitis Suppurativa Lesions are Characterized by Increased Kinase Signal-	No PAID syndrome

	ing Activity. Rumberger B.E., Boarder E.L., Owens S., Howell M.D. Journal of Investigative Dermatology (2019) 139:9 Supplement (S274).	
155	PAPA and FMF in two siblings: Possible amplification of clinical presentation? A case report. Maggio M.C., Ceccherini I., Grossi A., Gattorno M., Corsello G. Italian Journal of Pediatrics (2019) 45:1 Article Number: 111.	No anti-IL-1 treatment
156	North American clinical management guidelines for hidradenitis suppurativa: A publication from the United States and Canadian Hidradenitis Suppurativa Foundations: Part II: Topical, intralesional, and systemic medical management. Alikhan A. Et al. Journal of the American Academy of Dermatology (2019) 81:1 (91-101).	No PAID syndrome
157	Pharmacological development in hidradenitis suppurativa. Matusiak, Jemec G.B., Szepietowski J.C. Current Opinion in Pharmacology (2019) 46 (65-72).	No PAID syndrome
158	PSTPIP1-associated myeloid-related proteinemia inflammatory syndrome/PAMI syndrome: Case report and review of the literature. Mejbri M., Theodoropoulou K., Hofer M. Annals of the Rheumatic Diseases (2019) 78 Supplement 2 (977-978).	No anti-IL-1 treatment
159	Pyoderma gangrenosum - a guide to diagnosis and management. George C., Deroide F., Rustin M. Clinical Medicine, Journal of the Royal College of Physicians of London (2019) 19:3 (224-228).	No PAID syndrome
160	The role of interleukin-1b in pyoderma gangrenosum and the novel off label use of probenecid to target this cytokine<br>Australasian Journal of Dermatology (2019) 60 Supplement 1 (16-17).	No PAID syndrome
161	A novel pathogenic NCSTN variant in a large Australian pedigree affected with hidradenitis suppurativa. Vekic D., Cains G., Woods J., Moghaddas F., Masters S. Australasian Journal of Dermatology (2019) 60 Supplement 1 (94).	No PAID syndrome
162	Integrated multidisciplinary approach to hidradenitis suppurativa in clinical practice. Timila Touhouche A. International Journal of Women's Dermatology (2020) 6:3 (164-168).	No PAID syndrome
163	Pyoderma gangrenosum after cesarean section treated with skin graft: A case report. Shen J., Zhang W., Jiang X. Medicine (United States) (2019) 98:18 Article Number: e15380.	No PAID syndrome
164	Biologic and small-molecule medications in the management of pyoderma gangrenosum. McKenzie F., Cash D., Gupta A., Cummings L.W., Ortega-Loayza A.G. Journal of Dermatological Treatment (2019) 30:3 (264-276).	Review
165	Scoring pyoderma gangrenosum. Schreml S. British Journal of Dermatology (2019) 180:3 (438-439).	Review

166	Scoping review on the use of canakinumab and anakinra in interleukin-1 antagonist receptor deficiency. Hernandez Parada J., Ruano Ruiz J.A., Sáez-Torres De Vicente M., Gómez Arias P.J., Gómez García F., Isla Tejera B. European Journal of Hospital Pharmacy (2019) 26 Supplement 1 (A144-A145).	Review
167	Successful control of hidradenitis suppurativa with verapamil: a case report. Laroche M.-L., Teste M., Vanoost J., Geniaux H. Fundamental and Clinical Pharmacology (2019) 33:1 (122-124).	No PAID syndrome
168	Updated medical treatments of hidradenitis suppurativa, more options, less evidence. Gulliver W. Experimental Dermatology (2019) 28 Supplement 2 (5-6).	Review
169	Effectiveness of secukinumab (Anti-IL- 17 Monoclonal Ab) in hidradenitis suppurativa: An open study (17 cases). Becherel P.-A., Fougerousse A.-C., Reguiai Z. Experimental Dermatology (2019) 28 Supplement 2 (22-23).	No PAID syndrome
170	Systematic review of immunomodulatory therapies for hidradenitis suppurativa. Lim S.Y.D., Oon H.H. Biologics: Targets and Therapy (2019) 13 (53-78).	No PAID syndrome
171	Hidradenitis suppurativa: infection, autoimmunity, or both? Constantinou C.A., Fragoulis G.E., Nikiphorou E. Therapeutic Advances in Musculoskeletal Disease (2019) 11.	Review
172	Recent advances in managing and understanding pyoderma gangrenosum. Fletcher J., Alhusayen R., Alavi A. F1000Research (2019) 8 Article Number: 2092.	Review
173	Hidradenitis suppurativa/acne inversa: a practical framework for treatment optimization – systematic review and recommendations from the HS ALLIANCE working group. Zouboulis C.C. Et al. Journal of the European Academy of Dermatology and Venereology (2019) 33:1 (19-31).	No PAID syndrome
174	Biologics for hidradenitis suppurativa: An update. Włodarek K., Ponikowska M., Matusiak L., Szepietowski J.C. Immunotherapy (2019) 11:1 (45-59).	Review
175	Biologics and immunoglobulins in the treatment of pyoderma gangrenosum – analysis of 52 patients. Herberger K., Dissemond J., Brüggestrat S., Sorbe C., Augustin M.. JDDG - Journal of the German Society of Dermatology (2019) 17:1 (32-41).	No PAID syndrome
176	Dysregulated neutrophil responses and neutrophil extracellular trap formation and degradation in PAPA syndrome. Mistry P. Et al. Annals of the Rheumatic Diseases (2018) 77:12 (1825-1833).	Review
177	Pyoderma gangrenosum: A review for the gastroenterologist. Plumptre I., Knabel D., Tomecki K. Inflammatory Bowel Diseases (2018) 24:12 (2510-2517).	Review
178	Unconventional therapies for hidradenitis suppurativa. Marasca C., Annunziata M.C.,	Review

	Napolitano M., Fabbrocini G. Expert Review of Clinical Pharmacology (2018) 11:9 (879-887).	
179	Overview and update on biologic therapy for moderate-to-severe hidradenitis suppurativa. Porter M.L., Golbari N.M., Lockwood S.J., Kimball A.B. Seminars in Cutaneous Medicine and Surgery (2018) 37:3 (182-189).	Review
180	Successful therapy for pyoderma gangrenosum with a Janus kinase 2 inhibitor. Nasifoglu S., Heinrich B., Welzel J. British Journal of Dermatology (2018) 179:2 (504-505).	No PAID syndrome
181	Effectiveness of systemic treatments for pyoderma gangrenosum: a systematic review of observational studies and clinical trials. Partridge A.C.R., Bai J.W., Rosen C.F., Walsh S.R., Gulliver W.P., Fleming P. British Journal of Dermatology (2018) 179:2 (290-295).	No PAID syndrome
182	Drug-induced sarcoidosis in a patient treated with an interleukin-1 receptor antagonist for hidradenitis suppurativa. Friedman B.E., English J.C. JAAD Case Reports (2018) 4:6 (543-545).	No PAID syndrome
183	Severe refractory hidradenitis suppurativa: Treatment with ixekizumab, two case reports. Cotter C., Tobin A.M., O'Connor R., Gallagher C., Connolly M. British Journal of Dermatology (2018) 179 Supplement 1 (70).	No PAID syndrome
184	Use of anakinra in treatment-resistant pyoderma gangrenosum. Ng A., Aamer M., Warburton K.L., Montgomery R., Laws P. British Journal of Dermatology (2018) 179 Supplement 1 (71-72).	No PAID syndrome
185	A complex case of vulval pyoderma gangrenosum. Nguyen Y., Lowe P. Australasian Journal of Dermatology (2018) 59 Supplement 1 (4-5).	No PAID syndrome
186	An open-label extension study (OLE) of MABP1 targeting interleukin-1ALPHA for Hidradenitis suppurativa (HS). Argyropoulou M. Et al. Experimental Dermatology (2018) 27 Supplement 1 (9).	No PAID syndrome
187	Validation of the IHS4 score as an outcome measure for Hidradenitis Suppurativa (HS): Application in treatment with MABP1 targeting interleukin-1ALPHA. Kanni T., Argyropoulou M., Stecher M., Dinarello C., Simard J., Giamarellos-Bourboulis E. Experimental Dermatology (2018) 27 Supplement 1 (20).	No PAID syndrome
188	Diagnosis and treatment of hidradenitis suppurativa. Rashed I., Welsh J.S. JAMA - Journal of the American Medical Association (2018) 319:15 (1618).	No PAID syndrome
189	MABp1 Targeting IL-1α for Moderate to Severe Hidradenitis Suppurativa Not Eligible for Adalimumab: A Randomized Study. Kanni T. Et al. Journal of Investigative	No PAID syndrome

	Dermatology (2018) 138:4 (795-801).	
190	Pyoderma gangrenosum and Sweet syndrome: the prototypic neutrophilic dermatoses. Wallach D., Vignon-Pennamen M.-D. British Journal of Dermatology (2018) 178:3 (595-602).	No PAID syndrome
191	Arguments for a national questionnaire-based screening for hidradenitis suppurativa in Denmark. Riis P.T., Andersen P.L., Jemec G.B. Acta Dermatovenerologica Alpina, Pannonica et Adriatica (2018) 27:3 (115-120).	No PAID syndrome
192	The critical role of macrophages in the pathogenesis of hidradenitis suppurativa. Shah A., Alhusayen R., Amini-Nik S. Inflammation Research (2017) 66:11 (931-945).	No PAID syndrome
193	Recognizing syndromic hidradenitis suppurativa: a review of the literature. Gasparic J., Theut Riis P., Jemec G.B. Journal of the European Academy of Dermatology and Venereology (2017) 31:11 (1809-1816).	Review
194	Hidradenitis suppurativa treatment with biological. Sanabria V.P.E., Piva M.M.M., Galimberti R.L. International Journal of Dermatology (2017) 56:11 (1311).	No PAID syndrome
195	Approach to the management of patients with hidradenitis suppurativa: A consensus document. Alavi A Et al. Journal of Cutaneous Medicine and Surgery (2017) 21:6 (513-524).	Review
196	An infant with fever, pyoderma gangrenosum, osteitis, synovitis and oral ulcers: Immunologyand rheumatology collaboration in a novel autoinflammatory phenotype. Kourosh A., Bharath A., Rider N., Forbes L., Muscal E. Journal of Clinical Immunology (2016) 36:3 (243-244).	No PAID syndrome
197	Dosage Considerations for Canakinumab in Children With Periodic Fever Syndromes. Zhuang L. Et al. Clinical Pharmacology and Therapeutics (2019) 106:3 (557-567).	No PAID syndrome
198	A novel gene mutation associated with common variable immunodeficiency and pyoderma.<br>Joseph N., Hostoffer R., Tcheurekdjian H. Annals of Allergy, Asthma and Immunology (2018) 121:5 Supplement (S94).	No PAID syndrome
199	Successful treatment of pyoderma gangrenosum with anakinra in a patient with Wiskott–Aldrich syndrome. Mercuri S.R., Paolino G., De Flammineis E., Didona D., Brianti P. Dermatologic Therapy (2018) 31:2 Article Number: e12582.	No PAID syndrome
200	Therapieoptionen beim Pyoderma gangraenosum. Quist S.R., Kraas L. Journal der Deutschen Dermatologischen Gesellschaft = Journal of the German Society of Dermatology: JDDG (2017) 15:1 (34-41).	No English

201	The role of interleukin-17 in the pathogenesis of hidradenitis suppurativa. Yao Y., Thomsen S.F. Dermatology Online Journal (2017) 23:7.	No PAID syndrome
202	Industry update: The latest developments in the field of therapeutic delivery, 1-31 December 2018. Timmins P. Therapeutic Delivery (2019) 10:4 (215-226).	No PAID syndrome
203	Combining omalizumab with another biotherapy. Fougerousse A.-C., Becherel P.-A., Pallure V., Boyé T., Reguiai Z., Gabison G., Barthelemy H., Badaoui A., Mahé E., Bulai Livideanu C. Acta Dermato-Venereologica (2019) 99:4 (448-449).	No anti-IL-1 treatment
204	One decade real life experience with anti-IL1 therapy in monogenic and multifactorial autoinflammatory diseases. Mendonca L.O. Et al. Pediatric Rheumatology (2019) 17 Supplement 1.	No PAID syndrome
205	Two genes and one phenotype: Same therapy? Ricci S. Et al. Pediatric Rheumatology (2019) 17 Supplement 1.	No PAID syndrome

Table S4. Mapping studies on PAPA.

ID article	Journal / Journal speciality/Year	Number of authors	Type of publication	Country	Type of study/subtype	Patients	Affiliation s	Uni or multicenter/ specialities involved	A priori design / Registered protocol	Funding (type) / Conflict of interest
1	Rheumatology/ Rheumatology/ 2005	4	Letter	The Netherlands	Observational/ Case report	1	1	Unicenter/ Rheumatology Immunology Pediatrics	No No	NA No
2	Pediatric Dermatology/ Dermatology/ 2005	3	Full paper	USA	Observational/ Case report	1	2	Unicenter/ Pediatrics Immunology Dermatology	No No	NA NA
3	British Journal of Dermatology/ Dermatology/ 2009	5	Full paper	Germany	Observational/ Case report	1	2	Unicenter/ Dermatology Rheumatology Internal Medicine	No No	NA No
4	Annals of Rheumatology Diseases/ Rheumatology/ 2011	6	Letter	The Netherlands	Observational/ Case report	3	2	Unicenter / Dermatology General Internal Medicine	No No	NA NA
6	JAMA Dermatology/ Dermatology/	9	Full paper	Austria	Observational/ Case report	1	7	Unicenter/ Dermatology, Immunology,	No No	NA No

ID article	Journal / Journal speciality/Year	Number of authors	Type of publication	Country	Type of study/subtype	Patients	Affiliations	Uni or multicenter/ specialities involved	A priori design / Registered protocol	Funding (type) / Conflict of interest
	2013							Laboratory Medicine, Pathobiology of the skin, Allergy		
8	The Journal of Rheumatology/ Rheumatology/ 2014	4	Letter	Italy	Observational/ Case report	1	1	Unicenter/ Pediatrics	No No	NA NA
11	Pediatric Rheumatology/ Rheumatology/ 2015	11	Abstract	Italy	Observational/ Case series	6	3	Multicenter / Pediatrics Rheumatology	No No	NA NA
12	Pediatric Rheumatology/ Rheumatology/ 2015	8	Abstract	Italy	Observational/ Case series	5	7	Multicenter/ Pediatrics Rheumatology Dermatology	No No	NA NA
13	Pediatric Rheumatology/ Rheumatology/ 2015	6	Abstract	Bosnia and Herzegovina	Observational/ Case report	1	4	Multicenter/ Rheumatology Immunology Allergy Pediatrics Gastroenterology	No No	NA NA

ID article	Journal / Journal speciality/Year	Number of authors	Type of publication	Country	Type of study/subtype	Patients	Affiliation s	Uni or multicenter/ specialities involved	A priori design / Registered protocol	Funding (type) / Conflict of interest
								and Hepatology<br>Infectious diseases		
16	The Journal of Rheumatology/ Rheumatology/ 2016	6	Abstract	Canada	Observational/ Case report	2	3	Multicenter<br>/<br>Pediatrics<br>Rheumatology	No<br>No	NA<br>NA
17	Rheumatology/ Rheumatology/ 2016	14	Full paper	Italy	Observational/ Case report	13	5	Multicenter/<br>Rheumatology<br>Pediatrics	No<br>No	Yes (public)<br>Yes (Novartis)
19	Infection/ Infectious diseases/ 2017	4	Full paper	Germany	Observational/ Case report And review	1	3	Multicenter/<br>Infectious medicine Allergy	No<br>No	NA<br>No
22	Pediatric Radiology/ Pediatrics/ 2018	7	Full paper	Canada	Observational/ Case series	2	8	Multicenter<br>/<br>Radiology,<br>Pediatrics,<br>Rheumatology,	No<br>No	NA<br>Yes (Sobi and Novartis)
25	Annals of Rheumatology	7	Abstract	Italy	Observational/ Case series	7	2	Multicenter<br>/	No<br>No	NA<br>Yes (

ID article	Journal / Journal speciality/Year	Number of authors	Type of publication	Country	Type of study/subtype	Patients	Affiliations	Uni or multicenter/ specialities involved	A priori design / Registered protocol	Funding (type) / Conflict of interest
	Diseases/ Rheumatology/ 2019							Dermatology, Rheumatology, ImmuNology, Pediatrics		Abbvie, SOBI, Novimmune, Roche, Novartis, SaNofi, Pfizer)
26	Pediatric Rheumatology/ Rheumatology/ 2019	14	Abstract	Italy	Observational/ Case series	34	2	Multicenter/ Rheumatology Immunology	No No	NA No
29	Arthritis Rheumatoid/ Rheumatology/ 2012	8	Full paper	USA	Observational/ Case report	5	4	Multicenter/ Allergy Infectious diseases Oncology Genome	No No	Yes (public/private) Yes (Pfizer)

Abbreviations: NA, not available PA.

Table S5. Mapping studies on PAMI.

ID article / Syndrome	Journal / Journal speciality/Year	Number of authors	Type of publication	Country	Type of study/subtype	Number of patients	Number affiliations	Uni or multicenter/ specialities involved	A priori design / Registered protocol	Funding (type) / Conflict of interest
21<br>PAMI	British Journal of Dermatology / Dermatology / 2018	6	Letter	Switzerland	Observational/ Case report	1	3	Multicenter / Dermatology, Genetics, Rheumatology, Pediatrics	No<br>No	Yes (public)<br>No
23<br>PAMI	Annals of Rheumatology Diseases / Rheumatology / 2018	12	Abstract	Russia	Observational/ Case series	6	2	Unicenter/ Pediatrics, Molecular laboratory	No<br>No	NA<br>No
24<br>PASS	British Journal of Dermatology /Dermatology/ 2019	14	Letter	France	Observational/ Case series	9	9	Multicenter/ Dermatology, Genetics, Pathology, Gastroenterology, Rheumatology,	No<br>No	Yes (public)<br>Yes (AbbVie, Almirall, Amgen, Boehringer-Ingelheim, Celgene, Janssen, Leo Pharma, Lilly, Mylan, Novartis, Pfizer, Sun

ID article / Syndrome	Journal / Journal speciality/Year	Number of authors	Type of publication	Country	Type of study/subtype	Number of patients	Number affiliations	Uni or multicenter/ specialities involved	A priori design / Registered protocol	Funding (type) / Conflict of interest
										Pharmaceuti-cals, Bristol-Myers Squibb, MSD, Roche-Chugai, AstraZeneca, Grunenthal, Ipsen/Menarini, Savient, SaNofi Aventis, UCB, and grant support from Pfizer and L'Oreal)
32<br>PAMI	Clinical and Experimental Rheumatology	6	Case report	Italy	Observational / Case report	1	4	Multicenter / Rheumatology, Pediatrics, Genetics	No<br>No	NA<br>No
33<br>PAMI	Pediatric Rheumatology	9	Full paper	Brazil	Observational / Case series	1	5	Multicenter / Immunology, Allergy, Pediatrics, Rheumatology	No<br>No	No<br>Yes (Novartis)
34<br>PAMI	Frontiers in Medicine	9	Case report	Italy	Observational / Case report	1	5	Multicenter / Nephrology, Genetics, Immunology	No<br>No	Yes (public)<br>No
35<br>PAMI	Pediatrics	7	Case report	Italy	Observational / Case report	1	4	Multicenter / Hematology, Pediatrics,	No<br>No	No<br>No

ID article / Syndrome	Journal / Journal speciality/Year	Number of authors	Type of publication	Country	Type of study/subtype	Number of patients	Number affiliations	Uni or multicenter/ specialities involved	A priori design / Registered protocol	Funding (type) / Conflict of interest
								Genetics		

Abbreviation: NA, not available.

Table S6. Mapping studies on PASH.

Study [references]	Journal / Journal speciality/Year	Number of authors	Type	Country	Type of study/subtype	Number of patients	Number affiliations	Uni or multicenter/ specialities involved	A priori design / Registered protocol	Funding (type) / Conflict of interest
5	Journal American Academy of Dermatology / Dermatology / 2012	4	Full paper	Germany	Observational/ Case report	1	1	Unicenter/ Dermatology	No<br>No	No<br>No
10	British Journal of Dermatology / Dermatology/ 2015	5	Letter	Spain	Observational/ Case report	1	1	Unicenter/ Dermatology	No<br>No	No<br>NA
14	Journal European Academy Dermatology and Venereology / Dermatology/ 2014	7	Full paper	Germany<br>Austria	Observational/ Case report	1	4	Multicenter/ Dermatology<br>Rheumatology<br>Allergy	No<br>No	No<br>No
18	International Journal of Dermatology / Dermatology / 2017	5	Abstract	Ireland	Observational/ Case report	1	1	Unicenter / Dermatology	No<br>No	No<br>No
20	Journal of clinical and aesthetic dermatology	4	Full paper	USA	Observational/ Case report	1	1	Unicenter/ Dermatology	No<br>No	No<br>No

**Study [references]**	**Journal / Journal speciality/Year**	**Number of authors**	**Type**	**Country**	**Type of study/subtype**	**Number of patients**	**Number affiliations**	**Uni or multicenter/ specialities involved**	**A priori design / Registered protocol**	**Funding (type) / Conflict of interest**
	/ Dermatology/ 2017									
27	Australasian Journal of Dermatology / Dermatology / 2020	5	Letter	Australia	Observational/ Case report	1	3	Multicenter/ Dermatology	No<br>No	NA<br>Yes (Sun Pharma)
36	Journal of the American Academy of Dermatology	7	Case report	France	Observational / Case report	1	2	Unicenter / Dermatology	No<br>No	No<br>No

Abbreviation: NA, not available.

Table S7. Mapping studies on PASS, PAPASH, and PAC syndromes.

ID article/Syndrome	Journal / Journal speciality/Year	Number of authors	Type of publication	Country	Type of study/subtype	Number of patients	Number affiliations	Uni or multicenter/ specialities involved	A priori design / Registered protocol	Funding (type) / Conflict of interest
7<br>PAPASH	JAMA Dermatology / Dermatology / 2013	6	Letter	Italy	Observational/ Case report	1	4	Multicenter / Dermatology Pediatrics	No<br>No	NA<br>No
9<br>PAC	Clinical and Experimental Dermatology / Dermatology/ 2015	6	Full paper	Israel	Observational/ Case report	1	2	Unicenter/ Dermatology Pathology	No<br>No	NA<br>No
15<br>PASS	Dermatology / Dermatology / 2016	9	Full paper	Switzerland	Observational/ Case report	1	2	Unicenter/ Dermatology Rheumatology	No<br>No	No<br>No
24<br>PASS	British Journal of Dermatology /Dermatology/ 2019	14	Letter	France	Observational/ Case series	9	9	Multicenter/ Dermatology, Genetics, Pathology, Gastroenterology, Rheumatology,	No<br>No	Yes (public)<br>Yes (AbbVie, Almirall, Amgen, Boehringer-Ingelheim, Celgene, Janssen, Leo Pharma, Lilly, Mylan, Novartis, Pfizer, Sun Pharmaceuti-

ID article/Syndrome	Journal / Journal speciality/Year	Number of authors	Type of publication	Country	Type of study/subtype	Number of patients	Number affiliations	Uni or multicenter/ specialities involved	A priori design / Registered protocol	Funding (type) / Conflict of interest
										cals, Bristol-Myers Squibb, MSD, Roche-Chugai, AstraZeneca, Grunenthal, Ipsen/Menarini, Savient, Sanofi Aventis, UCB, and grant support from Pfizer and L'Oreal)
28 PASS	Journal European Academy Dermatology and Venereology / Dermatology / 2020	7	Letter	France	Observational/ Case report	1	3	Multicenter / Dermatology, Genetics		NA NA
30 PAPASH	Medicine / General Medicine / 2014	12	Full paper	Italy	Observational/ Case report	1	7	Multicenter/ Dermatology Rheumatology General Internal Medicine		Yes (public) No

Abbreviation: NA, not available.

Table S8. Genetics and clinical characteristics of patients with PAPA included in the review.

ID article/ ID Patient	Gender / age onset / age diagnostic / country	Number cycles	PG	PA	A	PSTPIP1 mutations	Others
19/1	Male<br>8 years<br>42 years<br>Germany	2	No	Yes	Yes	p.A230T	Low fever, cell count sinovial 54,000/microlitro, 82% neutrófilos, no cristales; Sinovitis right elbow, right knee, ankle, fingers and toes
17/2	Male<br>NA<br>4 years<br>Italy	1	No	Yes	No	p.E250Q	NA
17/3	Male<br>NA<br>12 years<br>Italy	2	Yes	No	No	p.E250K	One cutaneous absces, microcitic anaemia (Hb 7.4 mg/dl), splenomegaly, growth delay
17/4	Female<br>NA<br>23 years<br>Italy	1	Yes	Yes	Yes	p.E256G	NA
17/5	Female<br>NA<br>2 years<br>Italy	1	No	Yes	No	p.E256G	Sterile osteomyelitis, one palpebral oedema
17/6	Female<br>NA<br>51 years<br>Italy	1	No	Yes	Yes	p.E250Q	Dactylitis/tendinitis
16/7	NA<br>NA<br>NA<br>Canada	1	No	Yes	No	p.A230T	NA
16/8	NA<br>NA	1	No	Yes	No	p.G904A	NA

ID article/ ID Patient	Gender / age onset / age diagnostic / country	Number cycles	PG	PA	A	PSTPIP1 mutations	Others
	NA<br>Canada						
13/9	Male<br>2 years<br>16 years<br>Bosnia and Herzegovina	1	Yes	Yes	Yes	p.E250Q	Microcytic anaemia
11/10	Male<br>NA<br>NA<br>Italy	1	Yes	No	No	Wild Type	Cutaneous abscesses, pyogenic muscular abscess
8/11	Female<br>1,5 years<br>3 years<br>Italy	1	No	Yes	No	p.E250Q	Low grade fever, leukocytosis, chronic recurrent multifocal osteomyelitis
6/12	Male<br>Early childhood<br>22<br>Iran	2	Yes	Yes	Yes	p.G258A	Aphthous lesions in oral mucosa, fever, CRP 24 mg/dL, hypocromic microcytic anaemia
4/13	Female<br>18 years<br>33 years<br>The Netherlands	1	No	Yes	Yes	p.A230T	Abscesses, abdominal pain, spontaneous abortion, thrombosis, fever, increased CRP
3/14	Male<br>12 years<br>42 years<br>Germany	1	Yes	Yes	Yes	p.A230T	NA
2/15	Male<br>5 years 8 years<br>USA	1	Yes	Yes	Yes	PSTPIP1 (UM)	NA
1/16	Male<br>16 years<br>16 years	1	No	Yes	Yes	p.A230T	Mild anaemia, ESR 85mm/h, CRP 144 mg/l

ID article/ ID Patient	Gender / age onset / age diagnostic / country	Number cycles	PG	PA	A	PSTPIP1 mutations	Others
	The Netherlands						
29/17	Male<br>2 years<br>46 years<br>USA	1	Yes	Yes	Yes	p.A230T	Pthergy, pustules, sterile skin abscesses, severe colonic inflammation with dapsone
29/18	Male<br>4 months<br>10 years<br>Hispanic	1	Yes	Yes	No	p.E250Q	Sterile osteomyelitis, sterile skin abscesses, recurrent otitis
29/19	Male<br>8 years<br>18 years<br>USA	1	No	Yes	Yes	p.A230T	NA
29/20	Female<br>1 month<br>18 years<br>USA	1	Yes	Yes	No	p.E250K	LymphadeNopathy, spleNomegaly, thrombocytopenia, hemolytic anemia, pharyngeal papillomatosis, T cell large granular lymphocytosis
22/21	Male<br>5 years<br>15 years<br>Canada	1	No	Yes	No	PSTPIP1 (UM)	NA
22/22	Male<br>4 years<br>16 years<br>Canada	1	NA	Yes	NA	PSTPIP1 (UM)	CRP elevation, ESR elevation, leukocytosis

Abbreviations: PA, pyogenic arthritis; A, acne; PG, pyoderma gangrenosusm; p, protein; PSTPIP1 (UM), unspecified-mutation in PSTPIP1 gene; NA, not available.

Table S9. Genetics and clinical characteristics of patients with PAMI included in the review.

ID article/ ID Patient	Gender/age onset/age diagnostic/ country	Cycles	PG	PA	A	PSTPIP1 mutations	Others
21/5	Male<br>7 years<br>23 years<br>NA	1	Yes	Arthritis	Yes	p.E250K	Anaemia, neutropenia, mild thrombocytopenia and hepatosplenomegaly, CPR elevation, elevation of serum zinc, calprotectin and calgranulin C, osteomyelitis
23/6	No data<br>No data<br>No data<br>Russia	1	No data	No data	No data	p.E250K	anaemia, neutropenia, mild thrombocytopenia and hepatosplenomegaly, CPR elevation, elevation of serum zinc, calprotectin and calgranulin C, osteomyelitis
31 /8	Male<br>At birth<br>1 year<br>Russia	1	No	Arthritis	No	p.E250K	Vasculitis, paniculitis, ulcerative gastritis, nonactive colitis, hepatosplenomegaly, lymphadenopathy, fever, cytopenia, myocarditis, hemolytic anemia
31 /9	Female<br>2.5 months<br>0.5 years<br>Germany	2	No	No	No	p.E250K	Lympadenopathy, fever, cytopenia
31 / 10	Female<br>At birth<br>5.4 years<br>UK	1	No	Arthralgia	No	p.E250K	Scalp hair heterochromia, lentigines, photosensitivity, chronic diarrhea, hepato/spenomegaly, lymphadenopathy, fever, cytopenia, aseptic osteomyelitis

ID article/ ID Patient	Gender/age onset/age diagnostic/ country	Cycles	PG	PA	A	PSTPIP1 mutations	Others
32/23	Male<br>7 years<br>8 years<br>Italy	1	No	Yes	No	E250K	Hepatosplenomegaly<br>Leukopenia with neutropenia<br>Trilinear dysplasia<br>CRP elevation<br>ESR elevation<br>Blood zinc elevation<br>Calprotectin MRP8/14 elevation
33 /11	Female<br>At birth<br>4 years<br>Brazil	1	No	Arthritis	NA	p.E250K, c.748G > A	Diarrhea, palaness, mucosal bleeding, neutropenia, anemiathrombocytopenia, fever, non-specific macular skin rash, spleNomegaly, high levels of ESR, CRP and SAA, zinc and calprotectin
34 /12	Male<br>6 months<br>8 years<br>Italy	3	Yes	Asymmetrica l polyarthritis at large joints of lower limbs	Yes	p.E250K, c.748G > A	Hepatomegaly, splenomegaly, microcytic anemia, neutropenia, dyseruthropoiesis, high serum levels of zinc and calprotectin, growth hormone deficiency, proteinuria, microhematuria, hyperuricemia, high levels of CRP and SAA, focal segmental glomerulosclerosis, renal fibrosis
35 /13	Male<br>At birth<br>2 years<br>Italy	1	NA	NA	NA	p.E250K, c.748G > A	Recurrent pyelonephritis, normochromic normocytic anemia, hepatosplenomegaly, dysmorphic features, icteric skin, severe inguinal hernia, axial hypotonia, marked developmental delay, anisopoikilocytosis,

ID article/ ID Patient	Gender/age onset/age diagnostic/ country	Cycles	PG	PA	A	PSTPIP1 mutations	Others
							dyserythropoiesis, fever, lymphadenopathy, high levels of CRP, procalcitonin, ferritin, interleukin-18

Abbreviations: PA, pyogenic arthritis; A, acne; PG, pyoderma gangrenosusm; p, protein; c, cDNA; PSTPIP1 (UM), unspecified-mutation in PSTPIP1 gene; NA, not availble.

Table S10. Genetics and clinical characteristics of patients with PASH included in the review.

ID article/ ID Patient	Gender/age onset/age diagNostic/ country	Number cycles	PA	SH	A	PSTPIP1 mutations	Others
5 / 1	Male<br>27 years<br>34 years<br>Russia	1	Yes	Yes	Yes	Increased number of CCTG microsatellite repeats in the PSPTIP1 promoter region with heterozygosity	20-pack-year smoking, increase of leucocytes, thrombocites, CRP and GGT and serum amyloid A
10 / 2	Male<br>17 years<br>32 years<br>Spain	1	NA	Yes	NA	NA	Ex smoker, BMI 25,
14 / 3	Female<br>15 years<br>22 years<br>Turkey	1	Yes	Yes	Yes	NA	NA
18 / 4	Female<br>31 years<br>34 years<br>Ireland	1	Yes	Yes	Yes	NA	Depression, high BMI, hepatitis B carrier, ex smoker,
20 / 5	Female<br>37 years<br>27 years<br>USA	1	Yes	Yes	Yes	NA	BMI 42.93, stage IV chronic disease, type II diabetes mellitus, hypertension, obesity, depression
27 / 6	Male<br>NA<br>43 years<br>Australia	1	Yes	Yes	Yes	NA	NA
36 / 7	Male<br>Adolescence<br>59 years<br>France	1	Yes	Yes	Yes	No	NA

Table S11. Genetics and clinical characteristics of patients with PASS, PAPASH and PAC included in the review.

ID article/ ID Patient	Syndrome	Gender/age onset/age diagnostic/ country	Cycles	PG	AS	A	SH	UC	PSTPIP1 mutations	Others
15/2	PASS	Male<br>NA<br>32 years<br>Congo	1	Yes	Yes	Yes	Yes	-	NA	Hepatitis B infection and α-thalassemia, elevated CPR
24/3	PASS	Female<br>32 years<br>33 years<br>France	1	Yes	Undifferentiated spondylitis	NA	Yes	-	NA	Intermittent fever
28/4	PASS	Male<br>NA<br>20 years<br>France	1	Yes	Yes	Yes	Yes	-	Sequence variant in the NLRP3 gene was detected: p.Q703K	NA
34 /12	PAMI	Male<br>6 months<br>8 years<br>Italy	3	Yes	Asymmetrical polyarthritis at large joints of lower limbs	Yes	No	-	p.E250K, c.748G > A	Hepatomegaly, spleNomegaly, microcytic anemia, neutropenia, dyseruthropoiesis, high serum levels of zinc and calprotectin, growth hormone deficiency, proteinuria, microhematuria, hyperuricemia, high levels of CRP and SAA, focal segmental glomerulosclerosis, renal fibrosis
35 /13	PAMI	Male<br>At birth<br>2 years<br>Italy	1	NA	NA	NA	NA	-	p.E250K, c.748G > A	Recurrent pyelonephritis, Normochromic Normocytic anemia, hepatospleNomegaly, dysmorphic features, icteric skin, severe inguinal hernia, axial hypotonia, marked

										developmental delay, anisopoikilocytosis, dyserythropoiesis, fever, lymphadeNopathy, high levels of CRP, procalcitonin, ferritin, interleukin-18
7/1	PAPASH	Female<br>14 years<br>16 years<br>Moldavia	1	Yes	Arthritis	Yes	Yes	-	p.E277D missense mutation	NA
9/7	PAC	Male<br>30 years<br>33 years<br>Israel	1	Yes	-	Yes	-	Yes	mutation p.G403R	Recalcitrant pustular rash

Abbreviations: PA, pyogenic arthritis; A, acne; PG, pyoderma gangrenosusm; SH, supurative hidradenitis; SA, ankylosing spondylitis; PsA, psoriatic arthritis; UC, ulcerative Colitis; p, protein; c, cDNA; PSTPIP1 (UM), unspecified-mutation in PSTPIP1; NA, not availble.
